# Thermal Density Fluctuations and Polymorphic Phase Transitions of Ethane (C_2_D_6_) in the Gas/Liquid and Supercritical States

**DOI:** 10.1021/acs.jpcb.4c01422

**Published:** 2024-05-15

**Authors:** Vitaliy Pipich, Joachim Kohlbrecher, Dietmar Schwahn

**Affiliations:** † Jülich Centre for Neutron Science (JCNS) at Heinz Maier-Leibnitz-Zentrum (MLZ), Forschungszentrum Jülich GmbH, Garching D-85747, Germany; ‡ Laboratory for Neutron Scattering, Paul-Scherrer Institute, Villigen CH-5232 PSI, Switzerland; § Forschungszentrum Jülich GmbH, Jülich Centre for Neutron Science (JCNS-1), Wilhelm-Johnen-Straße, Jülich D-52428, Germany

## Abstract

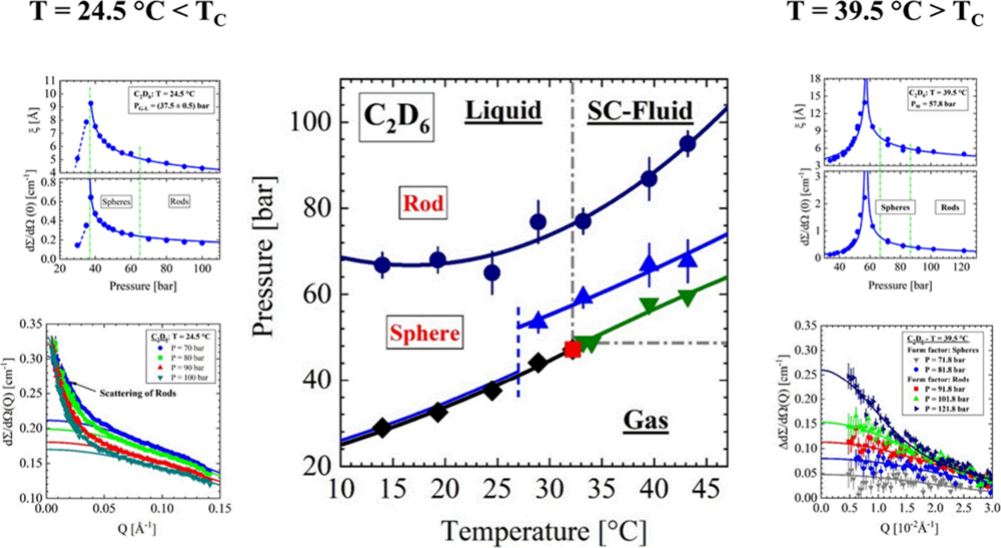

The phase behavior of the liquid C_2_D_6_ below and above the critical point was investigated using small-angle neutron scattering (SANS) in temperature and pressure ranges from 10 to 45 °C and 20 to 126 bar, respectively. The scattering of thermal fluctuations of the molecular density was determined and thus the gas–liquid and Widom lines. At the same time, we observed additional scattering of droplets of more densely packed C_2_D_6_ molecules above the gas–liquid line and in the supercritical fluid regime from just below the critical point for all temperatures at about Δ*P* = 10 bar above the Widom line. This line is interpreted as the Frenkel line. These results are consistent with our previous studies on CO_2_ and thus indicate a universal phase behavior for monomolecular liquids below and above the critical point. The interpretation of the Frenkel line as the lower limit of a polymorphic phase transition is in contrast to the usual interpretation as the limit of a dynamic process. The correlation lengths (ξ) of the thermal density fluctuations at the critical point and at the Widom line are determined between 20 and 35 Å and thus in the range of the droplet radius between 60 and 80 Å. These long-range fluctuations appear to suppress the formation of droplets, which can only form at about 10 bar above the critical point and the Widom line when ξ becomes smaller than 10 Å.

## 1 Introduction

In classical textbooks on thermodynamics such as ref (1), the pressure–temperature diagram of the phase diagram of low monomolecular liquids looks simple, as only the gas–liquid line is shown, which ends at the critical point. The gas–liquid line describes the location of first-order transition of the gas into the liquid phase, which becomes a second-order one at the critical point. No first-order phase transition is expected in the supercritical (SC) fluid regime at temperatures above the critical point. This view has changed in recent years, as can be read in a recently published overview of the history of this research^2^ and in the two most recent text books by Proctor and Maynard-Casely^3^ and Trachenko,^4^ in which the relevant boarder lines in the SC regime such as the Widom and Frenkel lines are extensively explained.

The Frenkel line was originally defined as a dynamic borderline between gas-like and liquid-like phases on the basis of purely diffusive and diffusive plus vibrational molecular motions, respectively.^5,6^ A similar borderline was proposed by Fisher and Widom, who suggested a “certain rough distinction between gas and liquid” based on the density pair correlation function, which shows a monotonic or oscillating asymptotic decay.^7^ A one-dimensional model assuming an infinite repulsion of the hard core and an attraction of the short-range square wells showed such a borderline, but this model could not be extended to higher dimensions.^8^ In two recent papers,^9,10^ we have determined the Widom line in CO_2_ with small-angle neutron scattering (SANS) starting from the critical point in the SC region from the maximum of the scattered intensity. This scattering of neutrons is determined solely by thermal fluctuations in CO_2_ density. Surprisingly, at higher pressure beyond the gas–liquid and Widom lines, we additionally observed the scattering from small spherical droplets, which transform into an elongated rod-like shape at higher pressure, allowing us to identify the Frenkel line and several polymorphic phase transition lines of yet unknown order.

**Figure 1:**
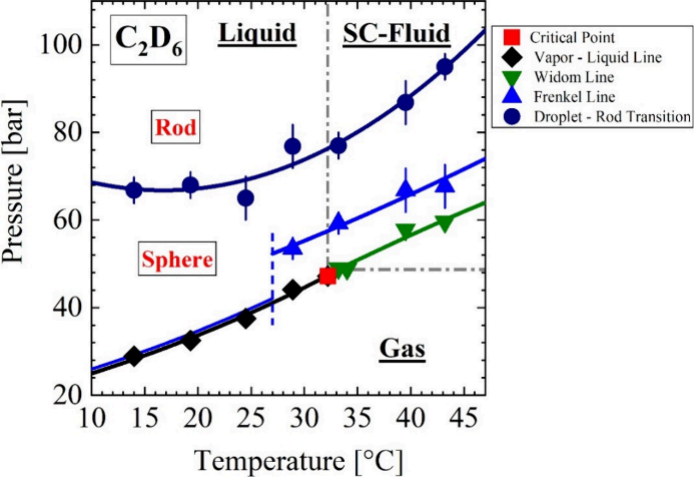
Pressure–temperature plane of the C_2_D_6_ phase diagram. Critical point at *T*_C_ = 32.2 °C, *P*_C_ = 47.2 bar. The ln *P* versus 1/*T* presentation of the phase diagram is depicted in Figure B1. Meaning of symbols: red square, critical point; black diamond, gas–liquid line; green inverted triangle, Widom line; blue triangle, Frenkel line; black circle, droplet–rod transition.

In the present work, we extended our investigations to another monomolecular liquid, namely, C_2_D_6_. We opted for the deuterated version of ethane because it has a much stronger scattering contrast, which is about 35 and almost 9 times larger than that of C_2_H_6_ and CO_2_, respectively (see Table 1 below). The results of our investigations are summarized in Figure 1, which shows the temperature–pressure projection of the phase diagram for C_2_D_6_ molecules. The critical point is observed at temperature and pressure values of *T*_C_ = 32.2 °C, *P*_C_ = 47.2 bar. Protonated ethane (C_2_H_6_) shows the same critical temperature but a slightly larger critical pressure of *P*_C_ = 48.7 bar (see below: Figure 4). The gas–liquid line and the Widom line follow the same line, which is only interrupted by the critical point.

**Table 1:** Parameters of Ethane-d6 (C_2_D_6_) Relevant for the Present SANS Experiments

molecule	molar mass [g/mol]	*b*_C_ [10^–12^ cm]	*d*Σ/*d*Ω_inc_ [cm^–1^]
C_2_D_6_	36.11	5.374	≃6.5 × 10^–3^
C_2_H_6_	30.7	–0.915	≃0.26
CO_2_	44.01	1.826	≃10^–6^ at 500 bar

The phase boundary of droplet formation coincides with the gas–liquid line at low temperatures and separates from the gas–liquid line (dashed blue line) between 25 and 30 °C to continue below *T*_C_ as the Frenkel line (blue line) about 10 bar above and parallel to the Widom line. We interpret the boundary of droplet formation, shown as blue triangles and line, as Frenkel line, in agreement with the CO_2_ case in ref (9). We believe that these observations are novel and in some way contradict its definition as a dynamic boarder line.^3,4^ At a larger pressure of about 30 bar above the gas–liquid and Widom lines, we observe a polymorphic change of the spherical droplets into an elongated rod-like shape in qualitative consistence with the CO_2_ fluid. In the following sections, the SANS data that led to the phase diagram in Figure 1 are presented and analyzed. They will be compared with data from literature leading to further conclusions about molecular liquids and fluids.

## 2 Methods

### Experimental Equipment

The neutron experiments were performed using a 40 m-long SANS-1 instrument from the continuous spallation neutron source SINQ at the Paul Scherrer Institute (PSI) in Switzerland. Sample-to-detector distances were 18 and 4.5 m with the corresponding collimator lengths of 18 and 6 m. The neutron wavelength was 6 Å with a wavelength resolution of Δλ/λ = 10% (FWHM). The temperature–pressure cell was especially designed for SANS experiments. Two sapphire windows with a diameter of 4 cm were used for the neutron passage and a thickness of 0.4 cm for the gas. This cell allows pressures of up to 500 bar. Temperature and pressure show an estimated absolute error of ±1 K and ±2 bar, respectively. The change in sample thickness for the gas is Δ*D*_S_ = 2.5 × 10^–4^ cm at a pressure of 100 bar, i.e., a negligible relative change of 6.3 × 10^–4^ if compared to the ambient thickness of 0.4 cm. The sample thickness is relevant for the absolute calibration of the scattering intensity, e.g., for determining the volume fraction of precipitates. The SANS data were corrected for background scattering and detector efficiency and were calibrated in absolute units using water as a secondary standard.

### 2.2 Sample

C_2_D_6_ was achieved from Eurisotop Cambridge Isotope Laboratories and had a 98% *D* concentration. Relevant parameters of C_2_D_6_ for the neutron scattering experiments are compiled in Table 1. The covolume of C_2_D_6_ molecules is related to the van der Waals parameter *b*_VdW_ and is approximately four times larger than the molecular volume Ω (ref (11) chapter 10.3).

The coherent scattering length *b*_C_2_D_6__ was determined from the corresponding values of carbon and oxygen given in ref (12) according to *b*_C_2_D_6__ = 2*b*_C_ + 6*b*_D_. The incoherent scattering *d*Σ/*d*Ω_inc_ evaluated for the molecular volume Ω at *T* = 28.9 °C and *P* = 60 bar is a negligible contribution to scattering. For comparison, we also give the corresponding parameters for ethane-h6 (C_2_H_6_) and CO_2_.

## 3 Results

Table 2 shows the investigated temperatures together with the corresponding determined pressures of the gas–liquid line (G-L), the Widom line (W), and the Frenkel line (F) as well as the transition line from spherical to rod-shaped domains (S-R), which are all shown in the phase diagram of Figure 1. Three temperatures are discussed in more detail in this section, namely, 24.5 °C, 28.9 °C, and the critical temperatures *T*_C_ = 32.2 and 39.5 °C, i.e., two temperatures below and one above the critical point (*T*_C_ = 32.2 °C, *P*_C_ = 47.1 bar) from reason, which becomes clear from the discussion of the phase diagram in Figure 1. The SANS data from the other temperatures mentioned in Table 2 are provided in the section of the Supporting Information.

**Table 2:** Parameters of the Pressure–Temperature Plane of the Phase Diagram of C_2_D_6_

*T* [°C]	*P*_G-L_ [bar]	*P*_C_ [bar]	*P*_W_ [bar]	*P*_F_ [bar]	*P*_S-R_ [bar]
14	28.9			28.9	66.8 ± 5
19.3	32.5			32.5	68 ± 5
24.5	37.5 ± 0.5			37.5 ± 0.5	65 ± 5
28.9	44.1 ± 0.5			53.5 ± 2.5	76.8 ± 5
32.2 (*T*_C_)	47.1			n.m.	
33.2			49 ± 0.1	59.3 ± 2.5	77 ± 3
34			48.85	n.m.	
39.5			57.8 ± 0.5	66.8 ± 5	86.8 ± 5
43.2			59.6 ± 1	67.7 ± 5	95 ± 5

### Temperature: 24.5 °C

**Figure 2:**
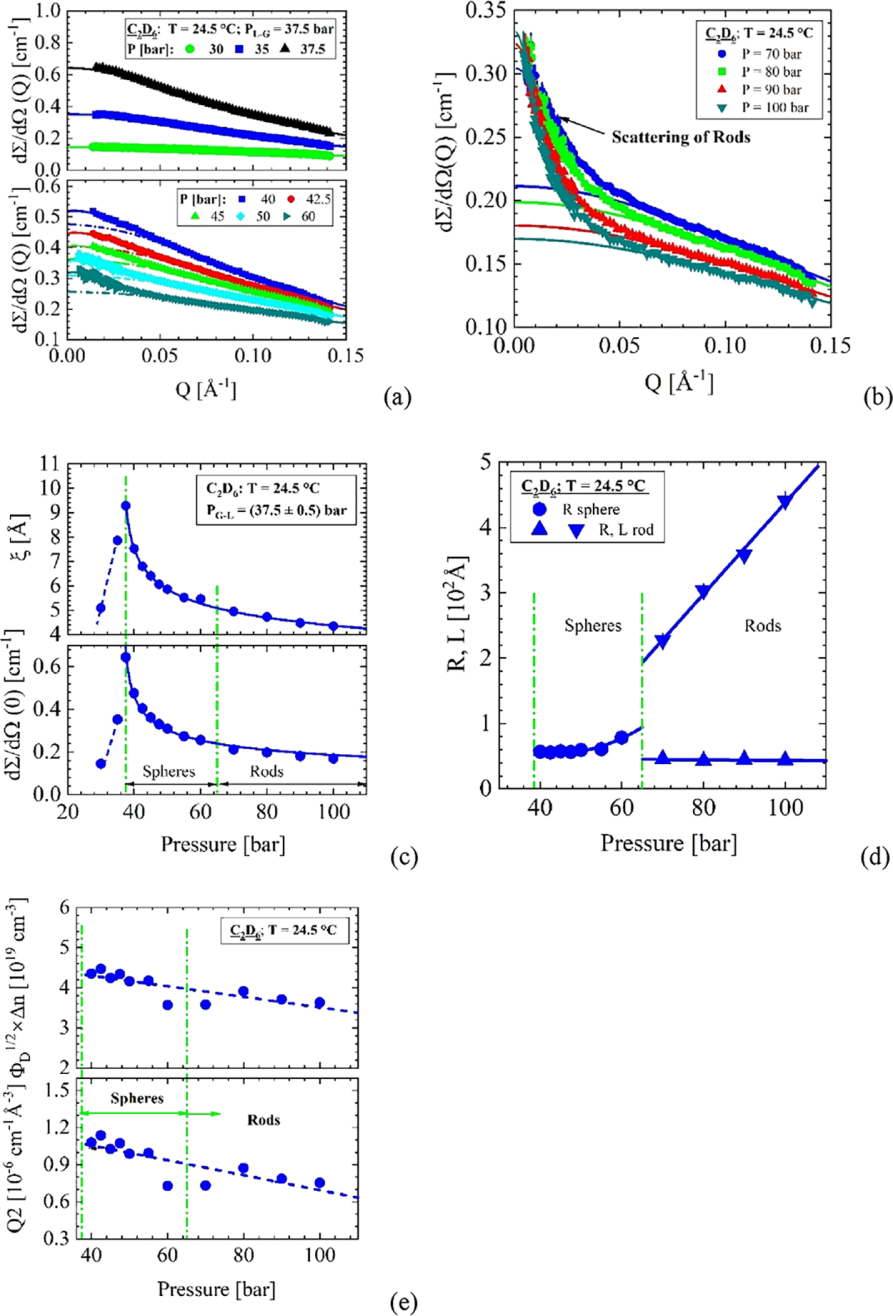
24.5 °C. (a, b) Scattering pattern *d*Σ/*d*Ω(*Q*) showing the thermal fluctuation as well as droplet part. (c) Correlation length and susceptibility (*d*Σ/*d*Ω(0)) of the density fluctuations below the critical temperature determining the gas–liquid line. The lines are guide for the eye even though fitted with eq A3. (d) Radius and length of the droplets indicate the two-phase area directly above the gas–liquid line. (e) *Q*2 as well as normalized with *n*(*T*,*P*) for C_2_H_6_ from ref (13).

The scattering curves measured at 24.5 °C are plotted in Figure 2a,b as a function of the momentum transfer *Q*. The upper part of Figure 2a shows the data of the gas phase below the gas–liquid line at 37.5 bar. *d*Σ/*d*Ω(*Q*) is determined by thermal density fluctuations and was analyzed using eqs A1 and A2, which provides two parameters, namely, the susceptibility (*d*Σ/*d*Ω(0)) in units of cm^–1^ and the correlation length ξ in units of Å. The correlation length is a measure of the specific extent of the fluctuations.^11^ Both parameters are depicted in Figure 2c versus pressure, clearly showing the G-L transition at 37.5 bar. The solid lines, as for all temperatures in this section, are guides for the eye resulting from fitting the power laws of eq A3, describing the critical behavior of susceptibility and correlation length becoming singular at the critical point (*T*_C_, *P*_C_). The two parameters are the critical amplitudes and isothermal critical exponents ν_T_ and γ_T_ of correlation length and susceptibility, respectively. The numerical values obtained for the critical exponents are discussed below only for the critical temperature *T*_C_ = 32.2 °C.

The lower part of Figure 2a shows above the G-L line, and this is the important observation of this manuscript, additional scattering at small *Q* caused by single spherical droplets (eqs A4 and A5). The spherical shape of the droplets changes to rod-like structures at a pressure of more than 70 bar, as can be seen from the analysis of the data in Figure 2b with the form factor for rod-like structures (eq A6). This analysis provides the droplet radius (*R*) and length (*L*) shown in Figure 2d showing slightly increasing radii from *R* = 55 to 80 Å and a relatively constant rod thickness of about 45 Å and an increasing rod length *L* from about 280 to 440 Å (Table S4).

Figure 2e shows the second moment (*Q*2, eq A9) of the droplet scattering Δ*d*Σ/*d*Ω(*Q*) as well as the product of the square root of the droplet volume fraction (Φ_D_) times the absolute values of the difference of the number densities (Δ*n* = |*n*_D_ – *n*_F_|) of the droplets (*n*_D_) and liquid (*n*_F_). The parameter Δ*n*^2^ is proportional to the scattering contrast, i.e., Δρ^2^ = (*b*_C_2_D_6__ × Δ*n*)^2^ of the droplets (eq A9). The ratio of *Q*2/(2π^2^[*b*_C_2_D_6__]_^2^_) ≃ Φ_D_ (Δ*n*)^2^ (eq A9) and therefore the product Φ_D_^1/2^ × Δ*n* in Figure 2e does not allow to determine the droplet volume fraction, as we do not know the difference of the number densities Δ*n*(*T*,*P*) of C_2_D_6_. In Appendix B2, we discuss this issue on the basis of some estimates of *n*(*T*,*P*) on the basis of C_2_H_6_.^13^ An interesting result is the formation of droplets in the liquid phase already starting at the G-L line, first as spherical droplets and then as rods at higher-pressure fields. This result confirms the results of earlier investigations on CO_2_^9^ and gives a first indication of a universal phase behavior in simple monomolecular liquids.

### 3.2 Temperature: 28.9 °C

**Figure 3:**
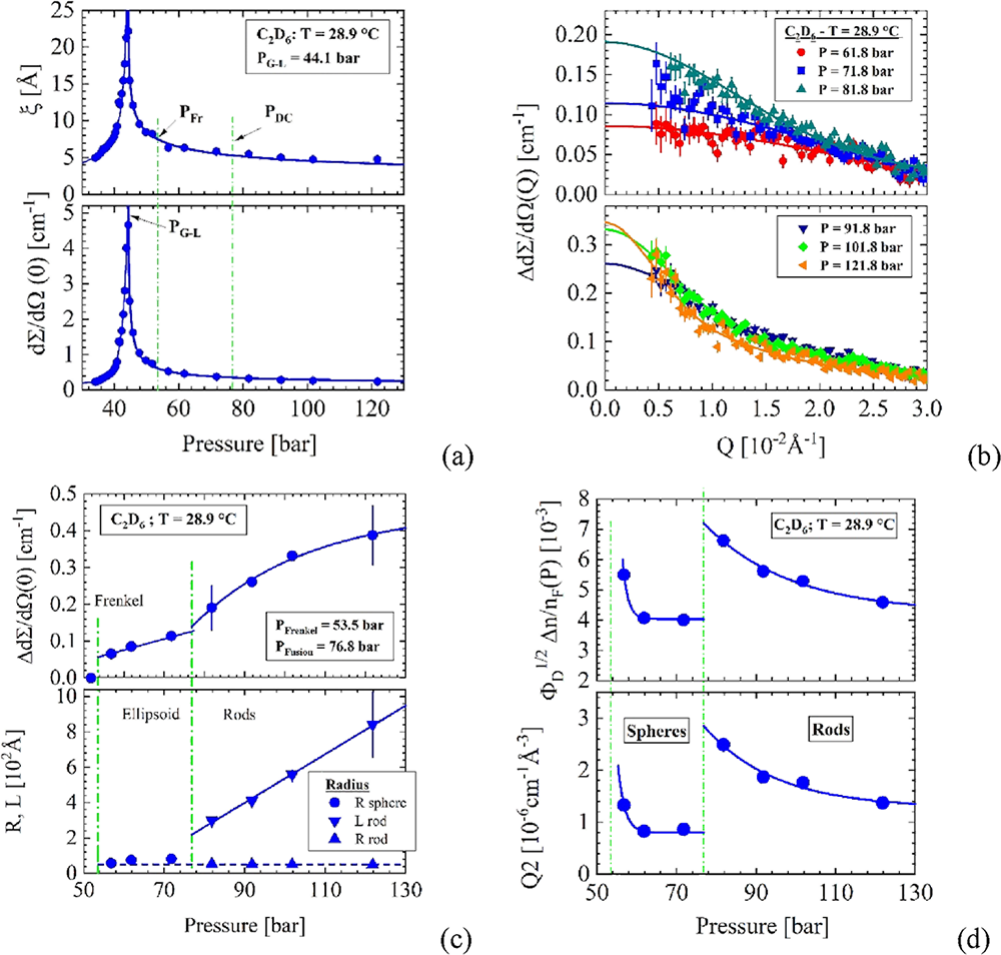
28.9 °C. (a) Correlation length and susceptibility *d*Σ/*d*Ω(0) of density fluctuations slightly below the critical temperature at 32.2 °C. The gas–liquid line is found at *P*_G-L_ = 44.1 bar. (b) Δ*d*Σ/*d*Ω(*Q*) represents the scattering from droplets obtained from the scattering pattern after subtraction *d*Σ/*d*Ω(*Q*) from thermal density fluctuations (eq A1). Scattering of droplets indicate the area above the Frenkel line. The upper curves were fitted with the spherical form factor (eq A5), the lower ones with the form factor of cylinders (eq A6). (c) The SANS parameters versus pressure derived from fitting *d*Σ/*d*Ω(*Q*)in (b). (d) *Q*2 as well as Φ_D_^1/2^ × Δ*n*/*n*_F_ normalized with *n*(*T*,*P*) for C_2_H_6_ from ref (13).

The susceptibility and the correlation length of the thermal density fluctuations along the isothermal path at 28.9 °C just below the critical temperature are shown in Figure 3a between 36 and 122 bar. The G-L line is determined at 44.1 bar slightly below the corresponding pressure of 44.4 bar of C_2_H_6_. Scattering by droplets is only observed at 53.5 bar and above, as can be seen from the cross-section Δ*d*Σ/*d*Ω(*Q*)of the droplet scattering in Figure 3b. The upper figure shows scattering from spheres whereas in lower one from rods. The parameters of the droplets are depicted in Figure 3c and d as Δ*d*Σ/*d*Ω(0), the dimensions of radius *R* and length *L* of rods as well as *Q*2 and Φ_D_^1/2^ × Δ*n*/*n*_F_, respectively. The radius of the spheres increases from about 58 to 82 Å, while the radius of the rods is fairly constant between 55 and 51 Å and their length increases from 300 to 752 Å (Table S5). The G-L line is clearly visible at 44.1 bar, while the start of domain formation now begins at the Frenkel line at 53.5 bar; i.e., at 28.9 °C (below *T*_C_ = 32.2 °C), we observe a clear separation between the G-L and Frenkel lines (see phase diagram in Figure 1). A separation of the G-L line and Frenkel line well below the critical point was also found for CO_2_ in (ref (9)Figure 1). This issue has been controversially discussed in the literature, as this makes a characteristic difference between the Widom and Frenkel lines, as the Widom line always starts at the critical point by definition.^3,21^

### 3.3 Critical Temperature: 32.2 °C

**Figure 4:**
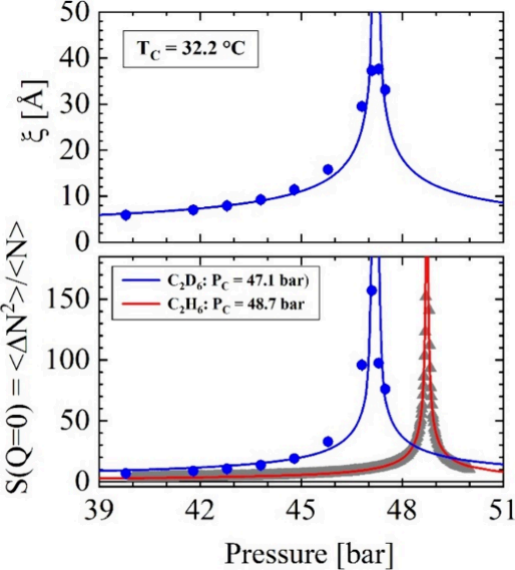
Susceptibility (*S*(*Q* = 0)) and correlation length (ξ) for the critical temperature when approaching the critical point along the isothermal path at *P*_C_ = 47.2 bar. The critical point of C_2_H_6_ is slightly higher at *P*_C_ = 48.75 bar, as derived for *S*(0) from *n*(*P*) of ref (13). The solid lines (blue and red) are adjustments of eq A3, which provide the critical amplitudes and exponents summarized in Table 3.

Figure 4 shows the correlation length (ξ) and the susceptibility (*S*(0)) of C_2_D_6_ at the critical temperature versus pressure as derived from the SANS scattering data (eq A1); the critical point is observed at *P*_C_ = 47.1 bar. Figure 4 also shows the susceptibility *S*(0) (gray points fitted by red line) of the C_2_H_6_ fluid derived from the number density *n*_F_(32.2 °C, *P*) obtained from the NIST data in ref (13), i.e., from ∂*n*∂*P*|_T,V_ applying eq A2. The C_2_H_6_ fluid shows a slightly larger critical value at *P*_C_ = 48.7 bar, as is shown from the peak position of *S*(0). We show *S*(0) only for the critical temperature after evaluation (better estimation) the corresponding scattering contrast, whereas for the other temperatures, the susceptibility is given in the units of *d*Σ/*d*Ω(*Q*) instead.

The power law fits of eq A3 are shown as solid lines in Figure 4, and their critical amplitudes and exponents are compiled in Table 3. The reason for this is that eq A3 is only applicable in the vicinity of the critical point (*T*_C_, *P*_C_) and the number density *n*(*P*,*T*) for C_2_D_6_ is not known. This is problematic, because the G-L line and Widom line of C_2_H_6_ and C_2_D_6_ are slightly different, which leads to noticeably different values, especially in the neighborhood of the transition lines. To reduce this discrepancy in the evaluation of *S*(0) of the C_2_D_6_ fluid at the critical temperature, we slightly shifted the *n*_F_(32.2 °C, *P*) distribution of C_2_H_6_ by Δ*P* = 1.6 bar to the critical pressure *P*_C_ of C_2_D_6_ of 47.1 bar. The critical amplitudes and exponents of C_2_H_6_ and C_2_D_6_ are the same within the error bars supporting our SANS data analysis on C_2_D_6_. However, the isothermal critical exponents differ substantially from the classical (mean field) and 3D Ising values.^14^ This could be related to the fact that we are approaching the critical point via the isothermal path. A study also performed on the basis of the NIST data along the isobaric path determined (isobaric) critical exponents for *S*(0) of γ_P_ = 0.80 and 0.67 for *T* < *T*_C_ and *T* > *T*_C_, respectively (for finite systems).^15^ A systematic and more detailed study of this topic might be interesting, filling a gap as we did not find any experimental study on this issue.

**Table 3:** Isothermal Critical Amplitude and Exponents of Susceptibility and Correlation Length along the Isothermal Pathway of Critical Temperature *T*_C_ = 32.2 °C^a^

molecule	*T*_C_ [°C]; *P*_C_ [bar]	*S*(0); γ_T_	ξ [Å]; ν_T_
C_2_ H_6_	32.2; (48.73 ± 0.01)	*A*_0_ = 0.86 ± 0.05	
		γ_T_ = 0.74 ± 0.01	
C_2_ D_6_	32.2; 47.1	*A*_0_ = 2.2 ± 1.9	ξ_0_ = 2.58 ± 0.23
		γ_T_ = 0.72 ± 0.19	ν_T_ = 0.47 ± 0.03

a The C_2_H_6_ data were derived from the NIST Chemistry WebBook website.^13^

### 3.4 Temperature: 39.5 °C

**Figure 5:**
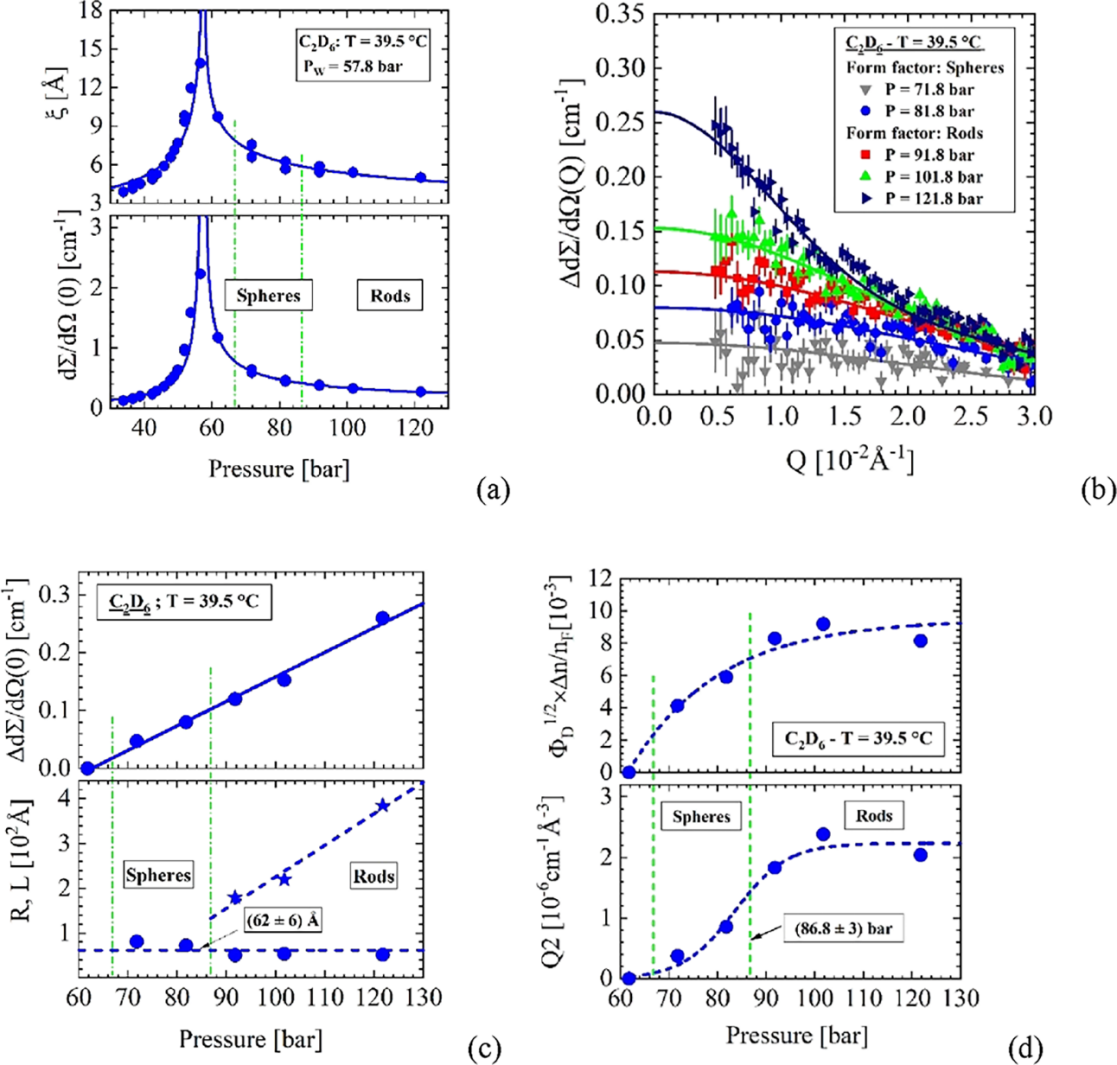
39.5 °C. (a) Correlation length and susceptibility (*d*Σ/*d*Ω(0)) of density fluctuations 7.3 K above the critical temperature. The maximum *d*Σ/*d*Ω(0) thereby determines the Widom line *P*_W_ = 57.8 bar. (b) Δ*d*Σ/*d*Ω(*Q*) represents the scattering from droplets indicating the area above the Frenkel line. (c) The SANS parameters versus pressure derived from fitting eqs A5 and A6. (d) *Q*2 as well as Φ_D_^1/2^ × Δ*n*/*n*_F_ normalized with *n*(*T*,*P*) for C_2_H_6_ from ref (13).

The scattering results measured at 39.5 °C are depicted in Figure 5.

Figure 5a shows the results of the thermal density fluctuations, whose maximum values of *d*Σ/*d*Ω(0) and ξ now determine the Widom line at 56.5 bar. The scattering data of Δ*d*Σ/*d*Ω(*Q*) in Figure 5b are from spherical and rod-shaped droplets above the Frenkel line. The evaluated droplet parameters in Figure 5c show a linear increase of Δ*d*Σ/*d*Ω(0), spherical droplets of radius between 80 and 70 Å, and rods with *R* = 52 to 56 Å and *L* increasing from 165 Å to nearly 390 Å above 87 bar (Table S7). Figure 5d confirms the increase of scattering intensity indicated by *Q*2 and the increase of Φ_D_^1/2^ × Δ*n*/*n*_F_, which indicates the increase in droplet volume (*Q*2) and volume fraction by a factor of about 2 and 3.3, respectively, if a change in scattering contrast due to the densities of liquid and droplets is neglected.

## 4 Discussion

The key message of this work is the observation of spherical droplets with higher C_2_D_6_ molecular density just above the gas–liquid and Frenkel lines and their polymorphic transformation into rod-like structures at about 10 bar higher pressures, as shown in the phase diagram in Figure 1. The radius of the spherical droplets increases slightly with pressure between 60 and 80 Å, while the thickness of the rods is stable with a radius of about 50 Å and their length *L* increases with pressure from about 150 to 900 Å. These observations show that in this region of the phase diagram, there is no difference between liquids and SC fluids in terms of static properties. We have made similar observations for CO_2_, which indicates a universal behavior for low monomolecular liquids.^9,10^

When analyzing the SANS data, we considered the droplets as isolated particles and applied the scattering laws for spheres (eq A5), ellipsoids, and rods (eq A6) to interpret them. However, the droplets could also be considered as a randomly distributed nonparticulate two-phase system described by the Debye–Anderson–Brumberger (DAB) model.^16,17^ The corresponding scattering law (eq S1) is given in Section 3 of the Supporting Information and describes the (droplet) morphology as density fluctuations that follow the correlation function γ(*r*) = exp (−*r*/ξ_D_) with the correlation distance ξ_D_ (ref (28) (chapter 11)). The application of the DAB model and the form factors of ellipsoids and rods to Δ*d*Σ/*d*Ω(*Q*), measured at 28.9 °C and 121.8 bar (Figure 3), clearly favors the model of isolated rod-shaped particles.

The volume fraction of the droplets cannot be determined with SANS alone because we do not know the molecular number density of C_2_D_6_ and especially of the droplets, as outlined in Appendix B2. However, we tried to provide a fairly reliable estimate of the droplet volume fraction. As a basis, we consider the number densities (*n*_F_) of C_2_H_6_ such as depicted for 14 and 43.2 °C versus pressure in Figure B2a and b, respectively, and for 300 and 900 bar versus temperature in Figure B2c. These numbers were taken from the NIST Chemistry WebBook.^13^ Assuming a molecular density (*n*_D_) for the droplets as for 300 to 900 bar, the volume fraction of the droplets Φ_D_ is between 4 × 10^–4^ and 2 × 10^–3^ for a temperature and pressure of 33.2 °C and 101.8 bar, respectively (Figure B3a). This estimate shows that the droplets always form a low concentration of isolated units. The droplet volume fraction Φ_D_ of the other temperatures, assuming *n*_D_ at 900 bar, are depicted in Figure B3b as a function of the reduced pressure *P*/*P*_F_ (*P*_F_ pressure at the Frenkel line). We see a fairly universal behavior of Φ_D_ for the temperatures between 28.9 and 39.5 °C, i.e., for temperatures of the Frenkel line showing a maximum of Φ_D_ ≃ 4.5 × 10^–4^ at *P*/*P*_F_ = 1.82. Only the volume fraction Φ_D_ for the highest temperature of 43.2 °C does not correspond to the general trend, which could be due to the deviations already observed in the SANS data in Figure S6b,c.

**Figure 6:**
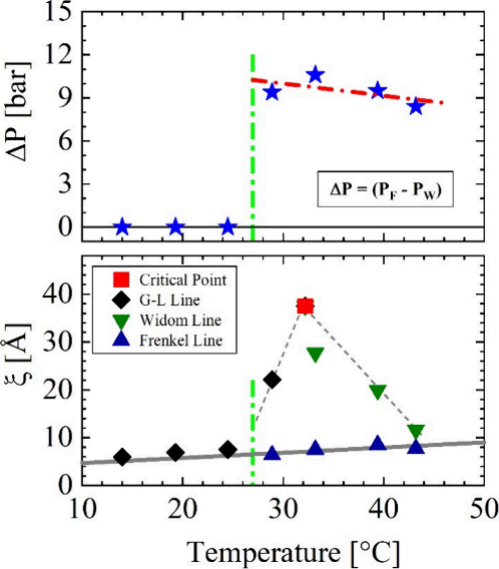
Correlation length of thermal density fluctuations of C_2_D_6_ fluid at the critical point, along the gas–liquid, Widom, and Frenkel lines compared with the difference of pressure between the gas–liquid/Widom and Frenkel lines. There appears a correlation between ξ and the onset of Frenkel line above 25 bar.

As already mentioned, we define the Frenkel line on the basis of the formation of small droplets. This view is supported by some recent theoretical studies on the “mesoscopic picture of the Frenkel line”, i.e., “not on the dynamics of individual atoms but on their instantaneous configurations” revealing a “percolation of solid-like structures (, which) occurs above the rigid–nonrigid crossover densities”.^18−20^ The course of the Frenkel line starts at the G-L line about 5 K below the critical point and runs about 10 bar above the Widom line without touching *T*_C_, as shown in Figure 1. In this context, it is interesting to compare the Frenkel line from SANS with the Frenkel line determined for C_2_H_6_ from Raman spectroscopy by Proctor et al.^21^ Both Frenkel lines have a qualitative similarity, namely their extension into the liquid region below the critical point (ref (12)Figure 6). On the other hand, the Frenkel line is determined for 300 K from Raman spectroscopy at about 2 kbar above the critical temperature, which corresponds to a pressure value about 35 times higher than our SANS value. This large discrepancy raises general questions about the interpretation of the Frenkel line, e.g., its definition as a dynamic boundary line between gas-like and liquid-like phases based on purely diffusive and diffusive plus oscillatory molecular motions. The formation of two phases above the Frenkel line from a predominantly liquid phase and a small volume fraction of droplets with denser molecular packing naturally raises the question of the mechanism of the C_2_D_6_ diffusion mechanism in the two phases.

The extension of the Frenkel line into the region below the critical point is observed for both C_2_D_6_ (Figure 1) and CO_2_ (ref (9)Figure 1) fluids and is discussed in detail in literature (ref (3) chapter 6.5). Strong thermal density fluctuations at and above the critical point (*T*_C_, *P*_C_) and the Widom line could be an explanation for the narrow range of the pure liquid phase between the Widom and Frenkel lines. The comparison of the correlation length ξ as a measure of the spatial extent of thermal fluctuations with the size of the droplets can be helpful and could influence the formation of droplets. The C_2_D_6_ and CO_2_ droplets respectively formed at the G-L and Frenkel lines show a radius between 60 and 80 Å and slightly smaller values between 30 and 40 Å (refs (8,9)). These values have to be compared with the correlation length ξ at the characteristic lines of the phase diagram (Figure 1), as compiled in Table 4 and depicted in Figure 6. The correlation length (ξ) at the G-L line (i.e., for *T* < 27 °C) and the Frenkel line is in the range between 6 and 8 Å, while for the G-L line (i.e., for *T* > 27 °C) and the Widom line, it is in the range between 20 and 40 Å. The upper Figure 6 shows a pressure difference, i.e., Δ*P* = (*P*_F_ – *P*_W_) of 9 to 11 bar between the Frenkel and Widom lines. There is therefore a correlation between the distance between the Frenkel line and the Widom line and the strength of the thermal density fluctuations (expressed by ξ in the lower Figure 6) compared to the size of the droplets. This observation could be interpreted to mean that the greater thermal fluctuations at the Widom line stabilize the liquid and thus prevent the formation of droplets, i.e., only allow their formation at a higher pressure at the Frenkel line. This interpretation is of course somewhat daring and must be verified by further SANS experiments, e.g., for higher temperatures when the thermal fluctuations become weaker. It also does not apply to the Proctor result of the Frenkel line, as this is too far away from the critical point.

**Table 4:** Parameters of the Pressure–Temperature Plane of the Phase Diagram of C_2_D_6_^a^

*T* [°C]	*P*_G-L_ [bar]	*P*_W_ [bar]	ξ_G-L;W_ [Å]	*P*_F_ [bar]	ξ_F_ [Å]	Δ*P* [bar]
14	28.9		5.94 ± 0.05	28.8	5.94 ± 0.05	0
19.3	32.5		6.92 ± 0.05	32.5	6.93 ± 0.05	0
24.5	37.5 ± 0.5		7.53 ± 0.05	37.5 ± 0.5	9.30 ± 0.05	0
28.9	44.1 ± 0.5		22.1 ± 0.05	53.5 ± 2.5	6.35 ± 0.06	9.4
32.2 (*T*_C_)	47.2 (*P*_C_)		37.5 ± 0.3			
33.2		49 ± 0.09	27.7 ± 0.04	59.3 ± 2.5	6.8 ± 0.17	10.3
39.5		57.8 ± 0.5	13.9 ± 0.1	66.8 ± 5	8.5 ± 0.05	9.5
43.2		59.6	11.6 ± 0.04	67.7 ± 5	7.73 ± 0.05	8.4

a Gas–liquid, critical temperature, and Widom lines.

In ref (3) (chapter 6), it is claimed that there is no “other first-order phase transition beyond the gas-liquid critical point until the melting point is approached”. This statement seems to contradict our results. Of course, the question remains whether the droplet phase transition above the Frenkel line is of first or second order, which we cannot yet answer from our SANS experiments.

**Figure B1:**
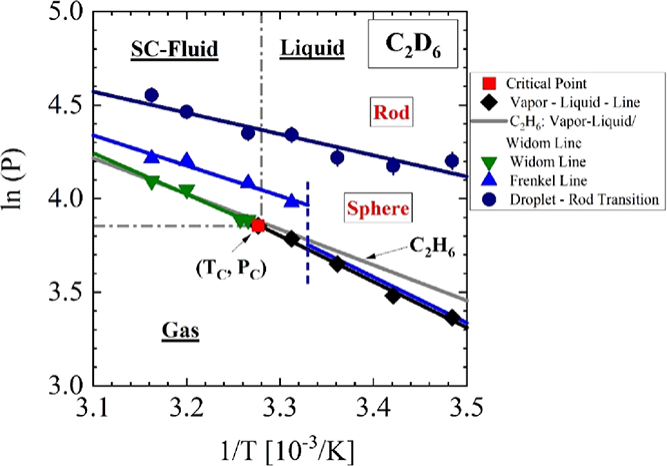
Pressure–temperature phase diagram of C_2_D_6_ in presentation of ln(*P*) versus 1/*T*. The gray line shows the corresponding gas–liquid and Widom lines of C_2_H_6_ showing a slightly smaller slope. Meaning of symbols: red square, critical point; black diamond, gas–liquid line; green inverted triangle, Widom line; blue triangle, Frenkel line; black circle, droplet–rod transition.

**Figure B2:**
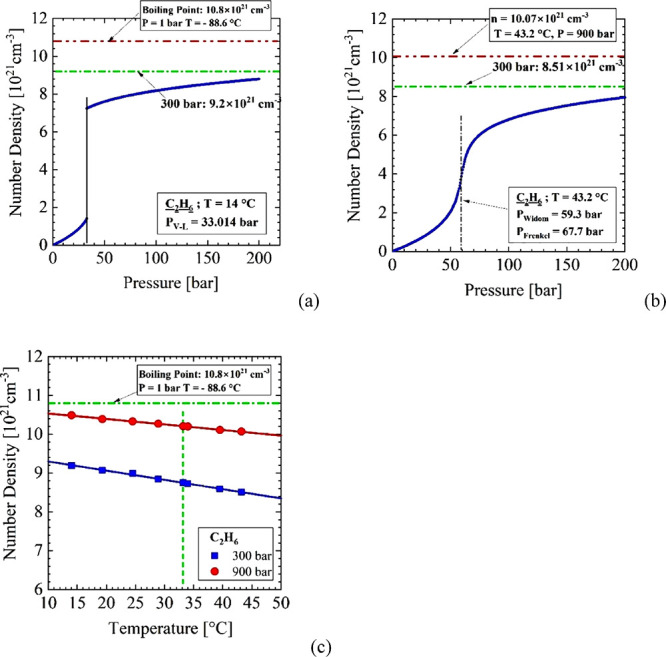
Number density of C_2_H_6_ at (a) *T* = 14 °C and (b) = 43.2 °C of the experimental range of pressure compared with higher pressure values of 300 and 900 bar as well as at the liquid boiling curve at 1 bar and −88.6 °C. These data were taken from the refs (13,33). Corresponding data from C_2_D_6_ are not available. (c) High-pressure (300 and 900 bar) number densities versus temperature of C_2_H_6_.

**Figure B3:**
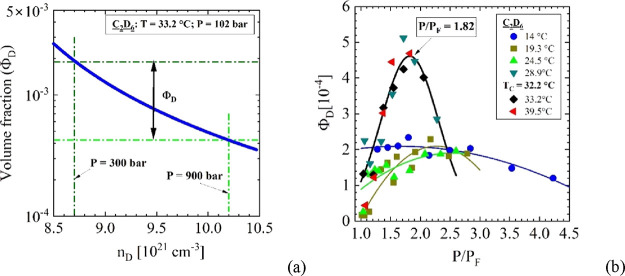
(a) Volume fraction Φ_D_ versus *n*_D_ as determined from *Q*2. The green dotted lines correspond to *n*_D_ for 33.2 °C at 300 and 900 bar. The volume fraction of droplets is between Φ_D_ 4 × 10^–4^ and 2 × 10^–3^. (b) Volume fraction of precipitates above the Frenkel line versus the normalized pressure *P*/*P*_Fr_. The volume fractions follow a universal line above 28.9 °C when the Frenkel line splits from the gas–liquid line. Only the 43.2 °C line deviates from this behavior.

The effect of the deuteration of ethane is illustrated in the phase diagram of Figure B1 this time plotted as ln(*P*) against 1/*T*. Almost the same critical point is known for C_2_H_6_, but its G-L line (gray line) has a slightly lower slope than that of the G-L line of C_2_D_6_. The G-L line is described by the Clausius–Clapeyron equation^29,30^ in eq B1, which assumes an ideal behavior of the gas. A latent heat (*L*) of (15.8 ± 0.8) kJ/mol and (20.4 ± 0.9) kJ/mol is absorbed during droplet formation in the C_2_H_6_ and C_2_D_6_ fluids, respectively (Table B1).

**Table B1:** Gas Phase of C_2_H_6_ Slightly More Stable than the C_2_D_6_ Gas Phase

fluid	*T*_C_ [°C]; *P*_C_ [bar]	*P*_0_ [kbar] (gas–liquid)	*L* [kJ/mol] (gas–Liquid)
C_2_D_6_	32.2; 47.2	146 ± 55	20.4 ± 0.9
C_2_H_6_	32.2; 48.9	24.3 ± 7.3	15.8 ± 0.8
CO_2_	31; 73.8	58.7 ± 2.4	16.9 ± 0.01

A secondary aspect of the present work relates to the analysis of the critical behavior, i.e., the determination of the susceptibility *S*(0) and correlation length ξ (eq A1) near the critical point (*T*_C_;*P*_C_) of the C_2_D_6_ and C_2_H_6_ fluids, as shown in Figure 4 and Table 3. Studies of several monomolecular fluids with SAXS are discussed by Chu in ref (22) and confirm the critical exponents of *S*(0) and ξ, as predicted by the 3D Ising model. Systematic SAXS studies on monomolecular SCFs for temperatures between 2 and 6% above the critical temperature have been carried out and published in recent years by Nishikawa and co-workers. Isothermal experiments on CO_2_ and CF_3_H are published in ref (23), and a more recent one on CO_2_ and methanol at *T*/*T*_C_ = 1.04 in ref (24). The isothermal experiments were carried out as a function of pressure, which was later converted into a numerical density using the corresponding equation of state (EOS) from the literature and is plotted as abscissa in the figures. The two maxima of the susceptibility S(0) and the correlation length ξ determine a so-called ridge, namely, the Widom line.

The susceptibility of C_2_H_6_ was calculated on the basis of *n*_F_ (*T*_C_ = 32.2 °C; *P*) in ref (13) using eq A2 and fitted with the corresponding power laws of eq A3, depicted as solid lines. The critical amplitudes and exponents are compiled in Table 3. The isothermal critical exponent of the susceptibility γ_T_ (the index *T* stands for the isothermal path along *T*_C_ = 32.2 °C) of both solutions is with γ_T_ ≃ 0.74 consistent within the error bars but does not follow the classical mean value γ_T_ = 1 or the 3D Ising behavior of γ_T_ between 1.2 and 1.3 (ref (11) Table 12.1).

A similar value of the isothermal exponent γ_T_ is derived for the isobaric critical exponent γ_P_ in ref (15), where, based on the NIST reference data (as we also do), a γ_P_ = *n*/(*n* + 1) with a “characteristic natural number” *n* = 2, i.e., γ_P_ = 2/3, for liquids at *T* > *T*_C_ is proposed for the isobaric approach to *T*_C_. Another paper^25^ predicts *n* = 1 for the phase transition of first order below *P*_C_ and for *n* = 2 a second-order transition at *P*_C_; the exponents corresponding to both singularities are 1/2 and 2/3, respectively. The observed deviation of the isothermal and isobaric critical exponents γ_T_ and γ_P_ from the classical values appears to be characteristic for the different paths to the critical point. These observations clearly show that further SANS experiments are needed to understand the critical behavior of monomolecular liquids, which in particular need to be carried out to the immediate vicinity of the critical point and should also include the approach along the critical isochore. For further details, see, e.g., chapter 4.4 of ref (26).

## 5 Conclusions

The present and previous studies on C_2_D_6_ and CO_2_ are a clear indication that droplet formation occurs in monomolecular liquids at higher pressures above and below the critical point.^9,10^ Droplet formation as a definition of the Frenkel line will lead to fundamental discussions about the meaning of the Frenkel line with regard to the concept of dynamic interpretation favored in the literature.^3,4^ In this context, the pressure experiments with Raman spectroscopy on C_2_H_6_ at 300 K could be interesting, in which the Frenkel line for 300 K was found at 2 kbar (200 MPa) representing a pressure about 40 times larger than our result of almost 50 bar.^21^ The formation of microscopic large droplets in monomolecular liquids and SC fluids in CO_2_ and C_2_D_6_ seems to be a new observation that probably was overlooked so far due to their small size in the range of 60 Å (6 nm) radius and estimated volume fraction between 4 × 10^–4^ and 2 × 10^–3^ (Appendix B2).

Further experiments with SANS and other methods are needed to shed more light on the obviously complicated structure of monomolecular fluids. In this context, the temperature and pressure range must be extended to higher values, and neutron spin–echo experiments could also provide interesting information on the dynamics of droplet behavior.^27^ We believe that the issue of static and dynamic properties with respect to the Frenkel line and their contradictory determination will lead to a fruitful discussion for a better understanding of phase behavior in SC fluids.

## APPENDICES

### Appendix A. Scattering Laws

In this section, we present the necessary scattering laws for our topic, which are reviewed in ref (28) and are also presented in ref (5) for SC–CO_2_. Thermal density fluctuations in monomolecular fluids such as C_2_D_6_ give rise to neutron scattering as described by the Ornstein–Zernike (OZ) law in eq A1. The corresponding differential macroscopic cross-section *d*Σ/*d*Ω(*Q*)represents the scattered

A1

intensity per unit volume in units of cm^–1^. In the case of isotropic scattering as in the present experiment, *d*Σ/*d*Ω(*Q*) is a function of the modulus of the scattering vector  determined as  from the neutron wavelength (λ) and the scattering angle (δ). The OZ law is determined by two parameters, namely, *d*Σ/*d*Ω(0) at *Q* = 0 and the correlation length ξ of thermal density fluctuations. The present experiment was carried out at constant volume (*V*) and as a function of pressure (*P*) along the isothermal path (*T*) (see phase diagram in Figure 1). This means that *d*Σ/*d*Ω(0) results from thermal fluctuations of the number density *n*(*T*,*P*) of the C_2_D_6_ molecules as shown in eq A2 for the relationship between the susceptibility S(0), dΣ/dΩ(0), and

A2

*∂n∂*P|_T,V_ representing the equilibrium limit of the fluctuation dissipation theorem as outlined in ref (11) (p 103 and 337) and in ref (29) (p 89). It has to be mentioned that the susceptibility *S*(0) in SANS experiments follow so-called elastic or static approximation, as there is no elastic scattering in liquids (ref (29) section 5). This is because the incoming neutrons of 6 Å have a kinetic energy of 2.27 meV (cold neutrons), which is about three orders of magnitude greater than the amount of energy (in the range of μeV) transferred in the scattering events. The contrast factor *K* = *n*(*T*,*P*) × [*b*_C_2_D_6__]^2^ for neutrons is determined by the product of *n*(*T*,*P*) and the square of the coherent scattering length *b*_C_2_D_6__ (Table 1). The susceptibility *S*(0) is without dimension that is determined for monomolecular gases and liquids by the mean square deviation of the number of molecules *N* in the irradiated volume (*V*_irr_) and the product of Boltzmann’s constant (*k*_B_), absolute temperature (*T*), and the first derivative of the number density *n*(*P*) (= <*N*>/*V*_irr_) in relation to pressure. This means that *S*(0) is determined on the basis of the number density *n*(*T*,*P*) at constants *T* and *V*, which is known from the determination of the EOS, as it emerges from the NIST data in ref (13). We have analyzed the correlation length ξ and the susceptibility *S*(0) along the isothermal pathway only for the critical temperature (*T*_C_) according to the power law of eq A3, which is fulfilled near the critical

A3

point of (*T*_C_; *P*_C_), where ξ and *S*(0) become singular at the critical point for macroscopic large volumes. The parameters are reduced pressure *p*, the critical amplitudes ξ_0_ and *A*_0_, and the isothermal critical exponents ν_T_ and γ_T_ (ref (11) section 12.7). Another approach to the critical point is along the isobaric pathway at the critical pressure *P*_C_ according to ξ ≃ ξ_0_*t*^–ν_P_^ and *S*(0) ≃ *A*_0_*t*^–γ_P_^ with *t* ≔ |*T*(*P*_C_) – *T*_C_|/*T*_C_. We did not carry out such experiments, as the determination of the critical exponents was not the original aim of our experiment.

Above the Frenkel line, we observe the formation of larger scattering units, i.e., Δ*d*Σ/*d*Ω(*Q*), which were identified as droplets (refs (9,10)) and analyzed according to eq A4 as the product of the

A4

scattering at *Q* = 0, Δ*d*Σ/*d*Ω(0), and the form factor *F*(Q) either of spheres of radius *R*_sp_ in eq A5

A5

or at higher pressure of randomly oriented rods of length *L*_rod_ and the radius of the cross-section *R*_rod_ as expressed in eq A6, respectively.^30^ The form factor of ellipsoids of revolution is the same

A6

as for spheres, however, with the radius  derived from the semi-axes *r*(*a*, ε, α = 0) = ε × *a* and *r*(*a*, ε, π/2) = *a* as derived from eq A7. The scattering ΔdΣ/dΩ(0)

A7

at *Q* = 0 is formulated in eq A8 (refs (28,30)) determining volume (*V*_D_) and volume fraction (Φ_D_) of

A8

the droplet phase. The strength of the scattering, i.e., the scattering contrast (Δρ^2^), is determined from the difference between the coherent scattering length densities of the droplets (*D*) and the liquid (*F*), i.e., Δρ = [ρ_D_ – ρ_F_] (ref (12)). For  is evaluated from the product of the coherent scattering length  (ref (12)) and the number densities (*n*) of C_2_D_6_ in the droplets (*n*_D_) and of the fluid (*n*_F_) phase delivering the expression Δρ =  × Δ*n* = ρ_F_ [Δ*n*/*n*_F_] or Δρ/ρ_F_ = Δ*n*/*n*_F_. Information about droplet volume fraction (Φ_D_) and the difference in molecular number density of droplet (*n*_D_) and fluid (*n*_F_), i.e., Δ*n* = [*n*_D_ – *n*_F_], can be obtained from the second moment of Δ*d*Σ/*d*Ω(*Q*), i.e., . According to eq A9, *Q*2 is approximately

A9

equal to the product of the droplet volume fraction Φ_D_ and the square of the number density Δ*n*^2^. An estimation of Δ*n* will be discussed in Appendix B2.

### Appendix B. Phase Diagram and Droplet Volume Fraction

In this section, we discuss the phase diagram of ethane C_2_D_6_, plotted as ln *P* versus 1/*T* in the context with the Clausius–Clapeyron equation, as well as the molecular densities in the droplets and the fluid for an estimation of the droplet volume fraction.

#### Appendix B1. Clausius–Clapeyron Equation

The representation of the C_2_D_6_ phase diagram of Figure 1 as ln(*P*) versus 1/*T* in Figure B1 is adapted to the Clausius–Clapeyron equation in the form of eq B1 describing the gas–liquid (G-L) line as a straight line whose slope is determined by

B1

the latent heat *L* via the gas constant *R* (= 8.314 J/(K mol)). The latent heat *L* of the gas–liquid transition refers to the product of temperature times the change of entropy per mole of the liquid to gas phase transition *L* = *T*Δ*S* with Δ*S* = (*S*_G_ – *S*_L_) (ref (11) p 110). Basically, Clausius–Clapeyron’s equation applies: *dP*(*T*)/*dT* = *L*/(Δ*V* × *T*) (ref (31) p 35) and (ref (1) p 288) with Δ*V* = (*V*_G_ – *V*_L_) the difference of the molar volumes of gas and liquid at the G-L line. The following assumption of *V*_G_ ≫ *V*_L_ and *V*_G_ according to the ideal gas law *V*_G_ = *R**T*/*P* (ref (32) p 250) leads via *d*(ln *P*(*T*)) = *L*/(*RT*^2^) *dT* to *P*(*T*) in eq B1. The latent heat (*L*) determined from the gas–liquid lines of C_2_H_6_ and C_2_D_6_ in Figure B1 are 15.8 and 19.7 kJ/mol, respectively, as recorded in Table B1. It shows that the C_2_D_6_ liquid requires about 30% more latent heat than C_2_H_6_ to transition to the gas phase. For comparison, *P*_0_ and *L* are also shown in Table B1 for CO_2_ (ref (5)Figure 1), showing similar values as ethane. The Widom and Frenkel lines also follow straight lines in the phase diagram and are thus described by an exponential function (solid lines).

#### Appendix B2. Number Density and Volume Fraction of Droplets

The difference of the coherent scattering length densities of the droplet (D) and fluid (F) phases observed beyond the G-L and Frenkel lines, i.e., Δρ = [ρ_D_ – ρ_F_] (ref (12)) determines the scattering intensity of the droplets. The scattering length density ρ of droplets and liquid is determined as ρ_D_ = *n*_D_ × *b*_C_2_D_6__ and ρ_F_ = *n* × *b*_C_2_D_6__ from the product of the coherent scattering length *b*_C_2_D_6__ (ref (12)) and the number densities *n*_D_ and *n*_F_ of the C_2_D_6_ molecules in the droplet (D) and the fluid (F) phases delivering the needed expression Δρ = *b*_C_2_D_6__ × Δ*n*. In particular, to determine the droplet volume fraction, we need information about the particle number densities Δ*n* as *Q*2/(2π^2^ [*b*_C_2_D_6__]_^2^_) (eq A9) is determined as the product of Φ_D_ Δ*n*^2^.

To our knowledge, the number density of C_2_D_6_ is not known. Therefore, we chose the number density of C_2_H_6_ gases and liquids from the NIST data as the best approximation instead (ref (13)), as shown in Figure B2a and b for the temperatures 14 and 43.2 °C, respectively, also selected in our experiments. Figure B2c shows the number density of C_2_H_6_ for 300 and 900 bar as a function of temperature and could be a reasonable estimate for the droplets observed in our experiments. The number density at a pressure of 900 bar is similar to the solid phase. As an example, Figure B3a shows the droplet volume fraction Φ_D_ versus *n*_D_ for *T* = 33.2 °C and *P* = 102 bar, which is determined from *Q*2 (Table S6) according to eq B2 with *n*_F_ = 7.366 × 10^21^ cm^–3^ and

B2

*Q*2/(2π^2^*b*_C_2_D_6__^2^) = 5.84 × 10^19^ cm^–6^. The volume fraction of droplets is estimated between Φ_D_= 4 × 10^–4^ and 2 × 10^–3^ indicated by the green dashed dotted lines in Figure B3a. Figure B3b depicts the droplet volume fraction Φ_D_ (assuming *n*_D_ at 900 bar) versus pressure, normalized by the pressure *P*_F_ at the Frenkel line. It is interesting to note that Φ_D_ in the presentation of Figure B3b follows the same values at the temperatures of 28.9, 33.2, and 39.5 °C, with a maximum at *P*/*P*_Fr_ = 1.82.

## Supporting Information Available

SANS figures not shown in the main text, i.e., the temperatures 14, 19.3, 33.2, 34, and 43.2 °C; tables with droplet parameters; and test of SANS data with scattering functions distinguishing between isolated particles and randomly distributed nonparticulate two-phase systems (PDF)
