# Supplementary material for: Thermal Density Fluctuations and Polymorphic Phase Transitions of Ethane (C2D6) in the Gas/Liquid and Supercritical States
Source: J Phys Chem B. 2024 May 15;128(20):5072–82. doi: 10.1021/acs.jpcb.4c01422 (PMC11129177; doi:10.1021/acs.jpcb.4c01422)
Supplement: Supplementary file 1 — jp4c01422_si_001.pdf [file jp4c01422_si_001.pdf]

Supporting Information

Thermal Density Fluctuations and Polymorphic Phase Transitions of Ethane (C_2D_6) in the Gas/Liquid and Supercritical State

Vitaliy Pipich[1], Joachim Kohlbrecher[2], Dietmar Schwahn[3,*]

1) Jülich Centre for Neutron Science (JCNS) at Heinz Maier-Leibnitz-Zentrum (MLZ), Forschungszentrum Jülich GmbH, D-85747 Garching, Germany.
2) Laboratory for Neutron Scattering, Paul-Scherrer-Institute, CH-5232 PSI Villigen, Switzerland
3) Forschungszentrum Jülich GmbH, Jülich Centre for Neutron Science (JCNS-1), Wilhelm-Johnen-Straße, D-52428 Jülich / Germany

*) Corresponding author:
Dietmar Schwahn (d.schwahn@fz-juelich.de)

Contents:

- SANS figures not shown in the main text, i.e. the temperatures: 14˚C, 19.3˚, 33.2˚C, 34˚C, and 43.2˚C.

- Tables with droplet parameters
- Test of SANS data with scattering functions distinguishing between isolated particles and randomly distributed nonparticulate two-phase systems.

1. SANS data not shown in the main text

Temperature: 14 °C.

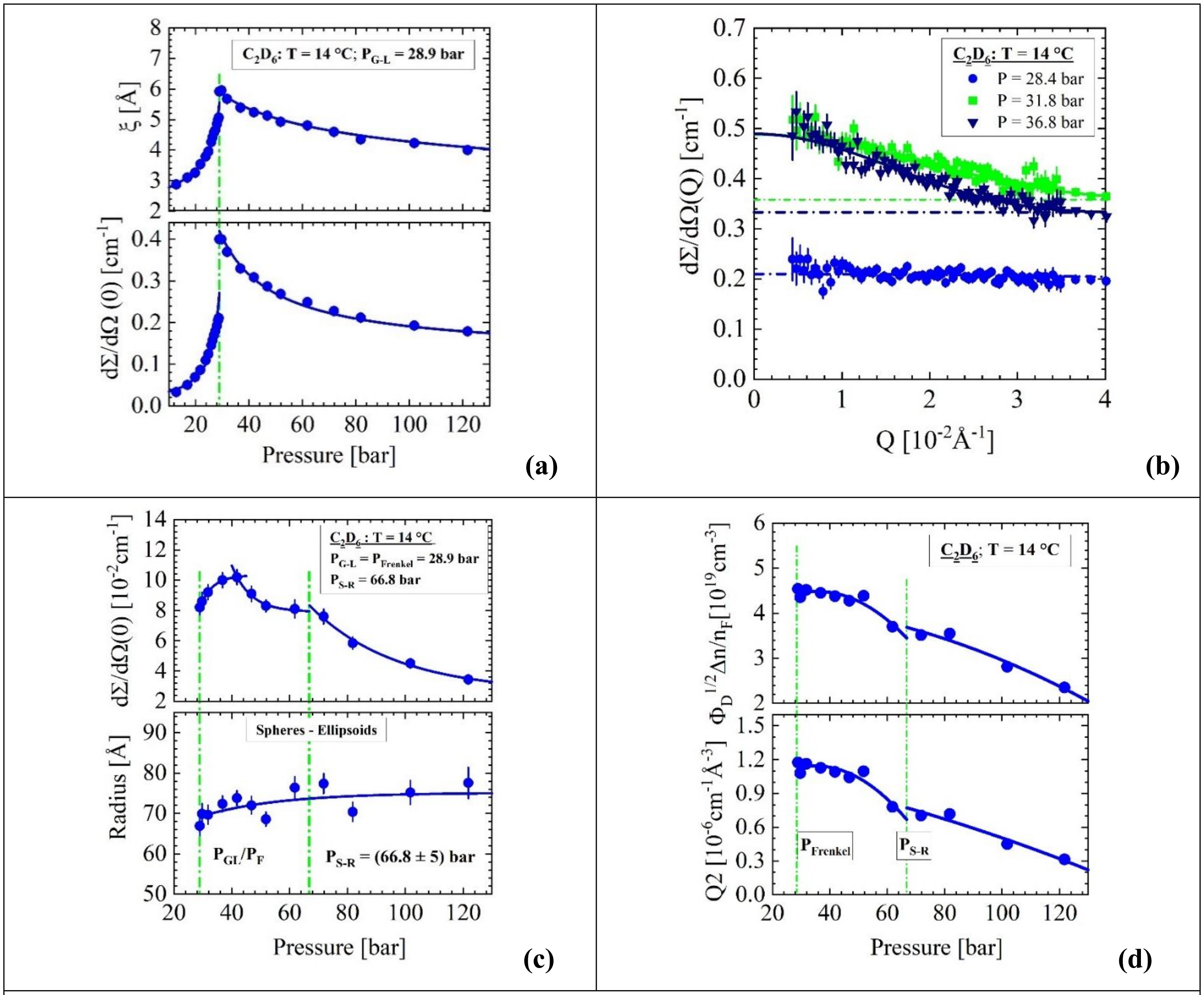

Figure S1. SANS data of 14 °C. (a) G-L transition of C_2D_6 slightly below C_2H_6. (b) Scattering pattern of 31.8 and 36.8 bar showing droplet scattering whereas 28.4 bar only thermal density fluctuations. The parameters in (c) and (d) show formation of spherical droplets above the G-L line.

Temperature: 19.3 °C.

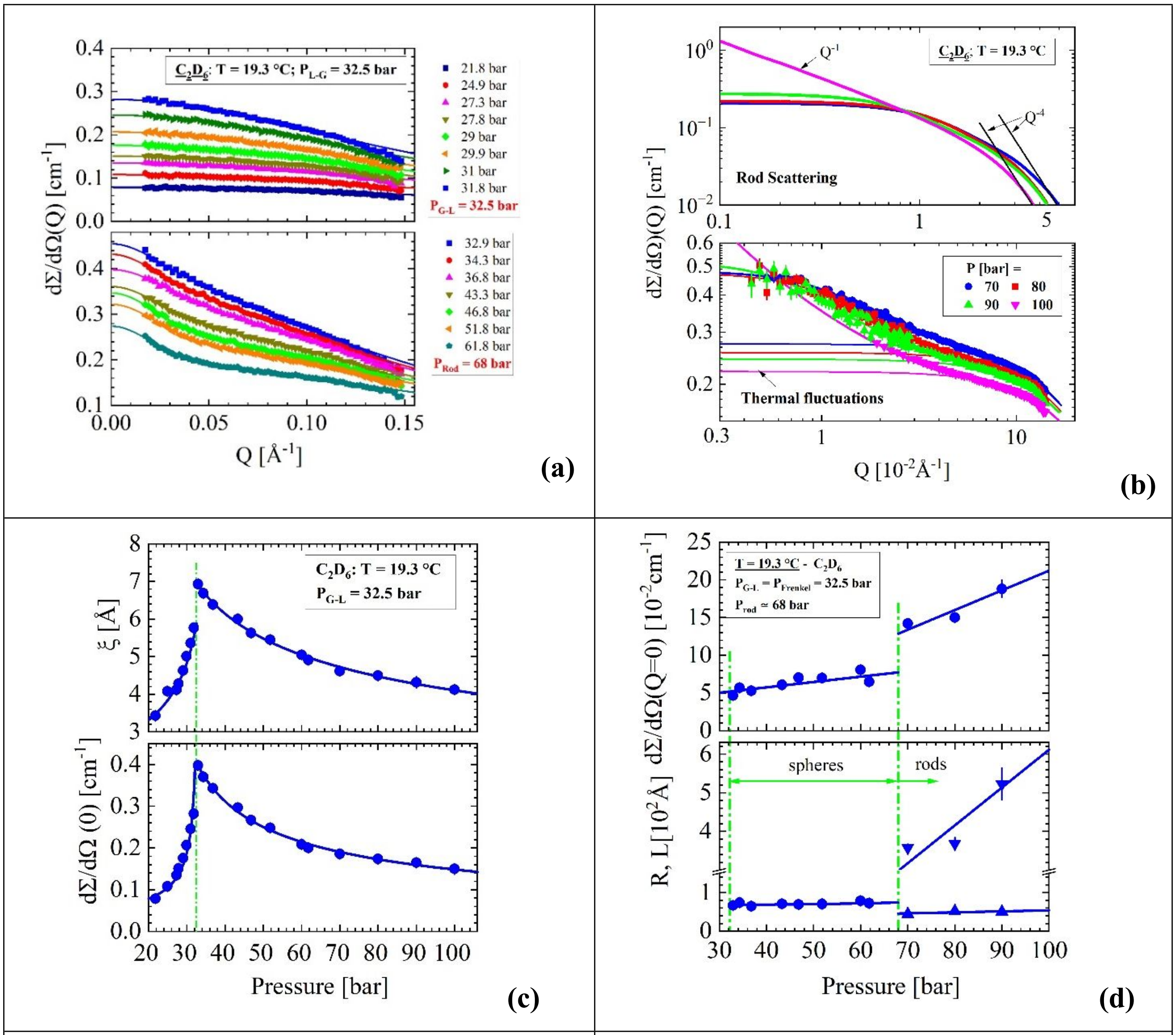

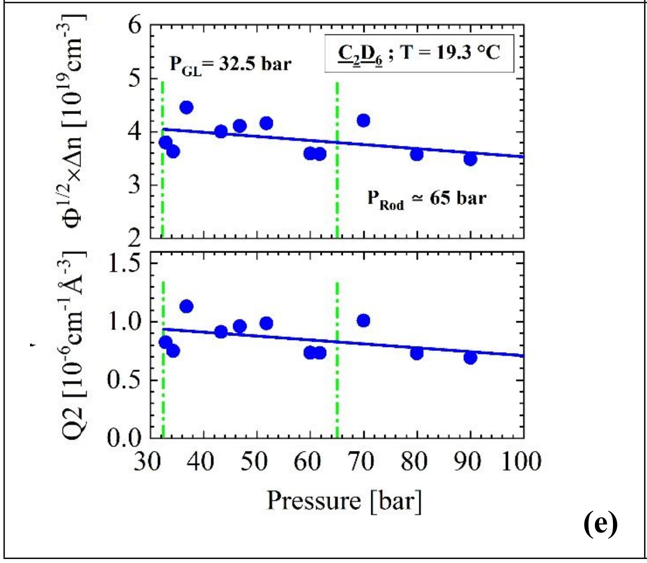

Figure S2. SANS data of T = 19.3 °C.
(a) SANS data below and above the G-L line. (b) scattering from rods. (c) to (e) Parameters determined from the scattering curves.

Temperature: 33.2 °C.

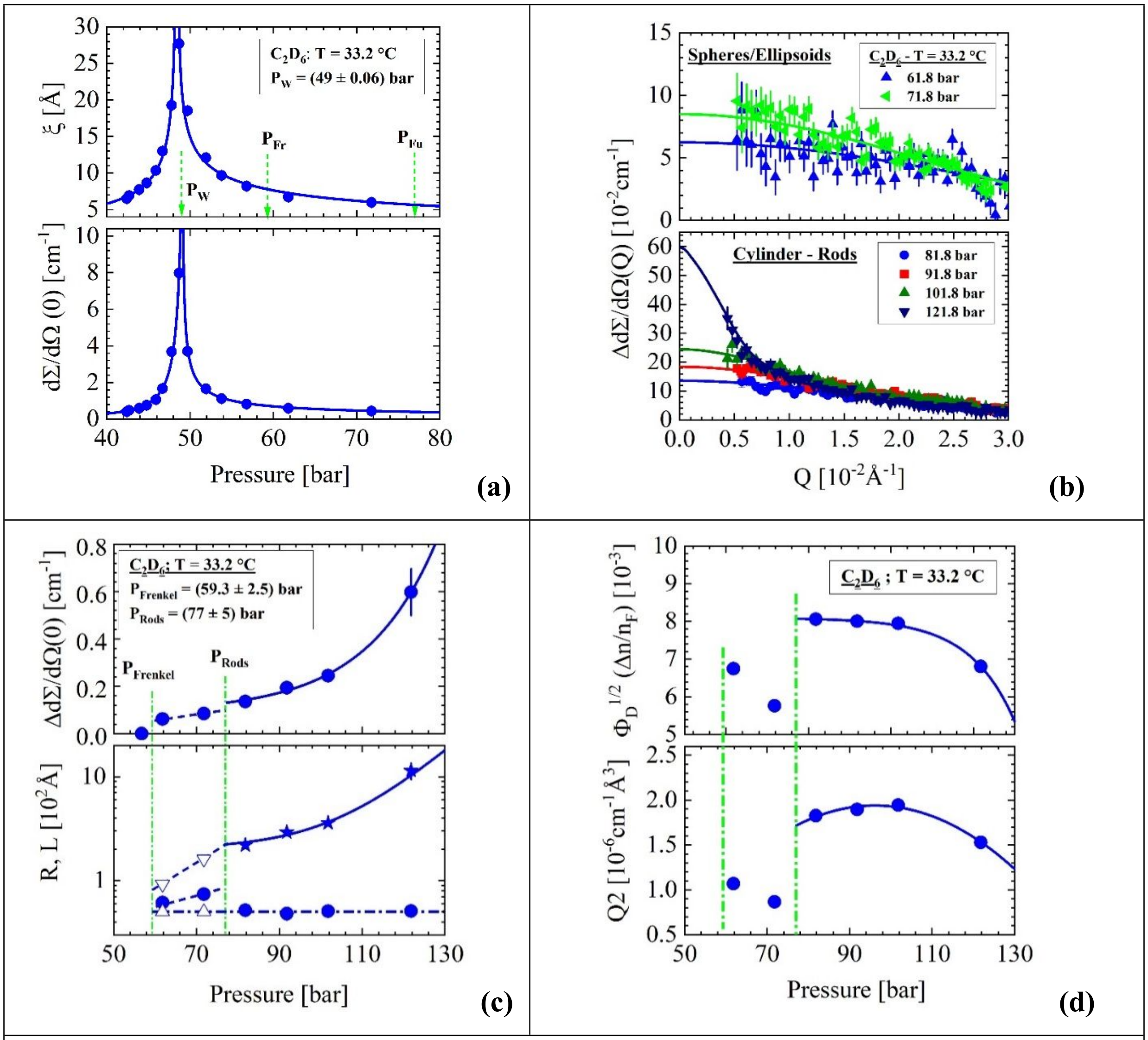

Figure S4. SANS data of 33.2 °C. (a) and (b) Scattering data versus pressure. (c) and (d) Parameters such as size and second moment Q2 of the droplets.

Table S1. Critical amplitudes and exponents of susceptibility and correlation length of along the isothermal pathway of temperature 33.2 °C.

Molecule	T [°C]; P [bar]	S(0); γ_-	S(0); γ_+	ξ_- [Å]; ν_-	ξ_+ [Å]; ν_+
C_2H_6	33.2 ; $P_- = 49.5 \pm 0.01$ $P_+ = 49.6$	$A_- = 0.99 \pm 0.001$ $\gamma_- = 0.70 \pm 0.001$	$A_+ = 0.60$ $\gamma_+ = 0.85$	--------	--------
C_2D_6	33.2; $P_- = 49.3 \pm 0.3$ $P_+ = 48.5 \pm 0.1$	$A_- = 2.26 \pm 0.21$ $\gamma_- = 0.69 \pm 0.05$	$A_+ = 1.29 \pm 0.07$ $\gamma_+ = 0.77 \pm 0.03$	$\xi_{0-} = 2.13 \pm 0.20$ $\nu_- = 0.6 \pm 0.05$	$\xi_{0+} = 4.65 \pm 0.10$ $\nu_+ = 0.36 \pm 0.01$

Temperature: 34 °C.

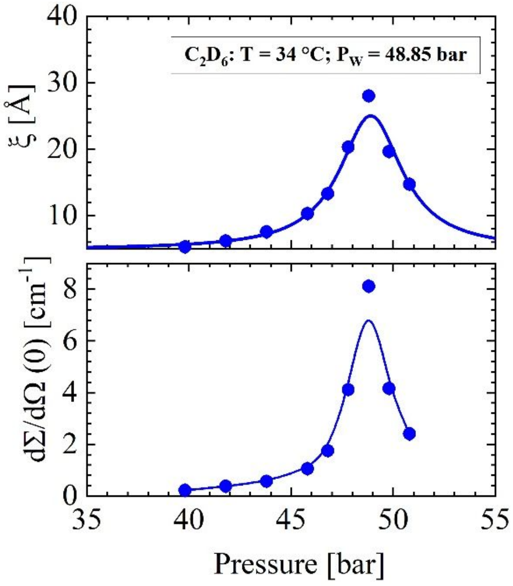

Figure S5. **34 °C**. Correlation length and susceptibility of C_2D_6. Both peaks correspond to the Widom line.

Temperature: 43.2 °C.

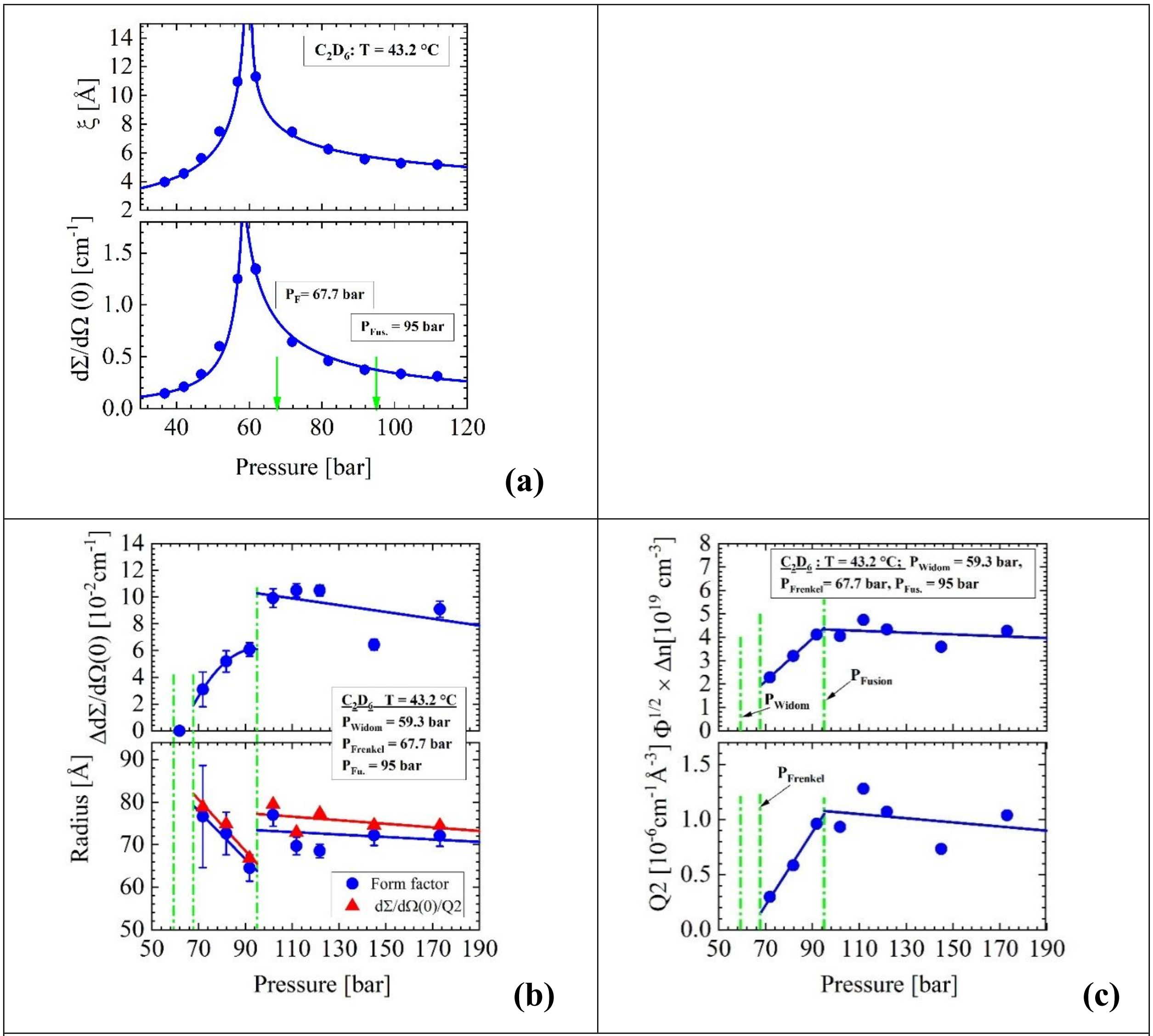

Figure S6. SANS data of 43.2 °C. (a) Susceptibility and correlation length, (b) and (c) parameters of the droplets.

2. Tables of droplet parameters

Table S2. Parameters from 14°C measurements from fit with spherical form factor. N_{C2H6} (900 bar) = 10.49×10^{21} cm^{-3}.

Pressure [bar]	S(0) [10^{-1} cm^{-1}]	ξ [Å]	ΔdΣ/dw(0) [10^{-2} cm^{-1}]	R [Å]	Q2 [10^{-7} cm^{-1} $Å^{-3}$]	n(C_2H_6) [10^{21} cm^{-3}]	$\Phi^{1/2}\times\Delta n$ [10^{19} cm^{-3}]	Φ [10^{-4}]
12.8	0.334±0.001	2.88±0.05	------	------	------	------	------	------
16.9	0.506±0.001	3.10±0.05	------	------	------	------	------	------
19.8	0.689±0.001	3.26±0.04	------	------	------	------	------	------
21.8	0.862±0.002	3.54±0.04	------	------		------	------	------
23.8	1.097±0.002	3.79±0.04	------	------	------	------	------	------
24.8	1.25±0.002	3.96±0.03	------	------	------	------	------	------
25.9	1.46±0.003	4.27±0.04	------	------	------	------	------	------
26.4	1.58±0.003	4.43±0.03	------	------	------	------	------	------
26.9	1.7±0.003	4.58±0.03	------	------	------	------	------	------
27.4	1.80±0.004	4.66±0.03	------	------	------	------	------	------
28.0	1.93±0.003	4.86±0.03	------	------	------	------	------	------
28.3	3.87±0.02	5.70±0.06	------	------	------	------	------	------
28.4	2.05±0.003	5.00±0.03	------	------	------	------	------	------
28.7	2.11±0.03	5.07±0.02	------	------	------	------	------	------
**28.8**	gas-liquid line - Frenkel line							
28.9	4.00±0.02	5.92±0.05	8.20±0.50	66.9±2.4	11.74	------	4.538	------
29.7	4.00±0.02	5.96±0.05	8.60±0.60	69.8±2.6	10.79	------	4.351	------
31.8	3.70±0.015	5.68±0.05	9.20±0.50	69.7±2.3	11.63	------	4.517	------
36.8	3.30±0.02	5.39±0.05	10±0.5	72.4±2.0	11.28	7.35	4.448	2.01
41.8	3.09±0.01	5.24±0.04	10.2±0.5	73.8±1.9	10.92	7.44	4.377	2.06
46.8	2.87±0.01	5.13±0.05	9.10±0.5	72±2.2	10.43	7.54	4.277	2.10
51.8	2.69±0.01	4.93±0.05	8.27±0.40	68.6±1.8	10.97	7.62	4.387	2.34

61.8	2.49±0.01	4.81±0.05	8.10±0.60	76.4±2.7	7.8	7.77	3.699	1.84
**66.8 ± 5**	Sphere (ellipsoid) – rod transition							
71.8	2.28±0.01	4.60±0.05	7.60±0.5	77.4±2.5	7.04	7.99	3.514	1.98
81.8	2.12±0.01	4.35±0.06	5.84±0.40	70.4±2.4	7.17	8.00	3.546	2.03
101.8	1.93±0.007	4.23±0.05	4.50±0.3	75.2±3.0	4.50	8.18	2.810	1.48
121.8	1.79±0.006	4.00±0.05	3.44±0.3	77.6±3.9	3.15	8.34	2.351	1.20

Table S3. Parameters of T = 19.3 °C determined with corresponding form factor. N_{C2H6} (900 bar) = 10.39×10^{21} cm^{-3}

Pressure [bar]	S(0) [10^{-1} cm^{-1}]	ξ [Å]	🖰dΣ/dw(0) [10^{-2} cm^{-1}]	R [Å]	L [Å]	Q2 [10^{-7} cm^{-1} $Å^{-3}$]	$Φ^{1/2}$×🖰n [10^{19} cm^{-3}]	Φ [10^{-4}]
21.8	0.789±0.003	3.43±0.08	------	------	------	------	------	------
24.9	1.084±0.004	4.08±0.07	------	------	------	------	------	------
27.3	1.35±0.003	4.12±0.04	------	------	------	------	------	------
27.8	1.51±0.003	4.28±0.04	------	------	------		------	------
29	1.76±0.004	4.63±0.05	------	------	------	------	------	------
29.9	2.07±0.004	5.01±0.04	------	------	------	------	------	------
31.0	2.46±0.004	5.36±0.03	------	------	------	------	------	------
31.8	2.82±0.005	5.77±0.03	------	------	------	------	------	------
**32.5**	gas-liquid line - Frenkel line							
32.9	3.98±0.02	6.93±0.05	4.67±0.60	67.0±4.6	------	8.23	3.80	0.17
34.3	3.70±0.01	6.69±0.04	5.70±0.50	73.8±3.6	------	7.50	3.627	0.16
36.8	3.43±0.02	6.39±0.05	5.30±0.40	64.6±3.0	------	11.31	4.454	0.26
43.3	2.97±0.01	6.00±0.06	6.09±0.50	71.1±3.3	------	9.13	4.00	1.45
46.8	2.67±0.01	5.63±0.06	7.05±0.42	69.5±2.4	------	9.61	4.106	1.63
50	2.45±0.05	5.47±0.08	7.41±0.18	81.7±1.9	------	6.02	3.25	1.08
51.8	2.48±0.01	5.45±0.05	7.00±0.35	70.6±2.1	------	9.86	4.159	1.80
60	2.09±0.01	5.05±0.05	8.08±0.25	78.5±1.6	------	7.35	3.591	1.49
61.8	2.00±0.01	4.91±0.05	6.50±0.34	72.5±2.2	------	7.32	4.033	1.93
**68±2**		Sphere (ellipsoid) – rod transition						
70	1.86±0.01	4.62±0.08	14.2±0.3	44.1±2.4	358±9	10.10	4.209	2.29
80	1.74±0.02	4.50±0.11	15.0±0.4	52.1±3.3	368±16	7.28	3.574	1.82
90	1.65±0.02	4.32±0.15	18.8±1.2	50.3±4.6	523±41	6.92	3.484	1.89
100	1.5±0.013	4.12±0.12	------	------	------	------	------	------

Table S4. Parameters of T = 24.5 °C determined with corresponding form factor. Parameters from N_{C2H6} (900 bar) = 10.33×10^{21} cm^{-3}

Pressure [bar]	S(0) [10^{-1} cm^{-1}]	ξ [Å]	🖰dΣ/dw(0) [10^{-2} cm^{-1}]	R [Å]	L [Å]	Q2 [10^{-7} cm^{-1} $Å^{-3}$]	$\Phi^{1/2}$×🖰n [10^{19} cm^{-3}]	Φ [10^{-4}]
30	1.45±0.003	5.09±0.03	------	------	------	------	------	------
35	3.52±0.007	7.86±0.03	------	------	------	------	------	------
37.5	6.44±0.02	9.28±0.04	------	------	------	------	------	------
38.5	gas-liquid line - Frenkel line							
40	4.75±0.02	7.53±0.05	4.77±0.24	56.7.0±2.3	------	10.8	4.35	0.26
42.5	4.04±0.01	6.8±0.03	4.04±0.19	55.5±1.6	------	11.38	4.47	1.32
45	3.62±0.01	6.41±0.03	4.70±0.18	57.5±1.6	------	10.27	4.24	1.29
47.5	3.30±0.01	6.06±0.04	4.67±0.20	56.4±1.8	------	10.74	4.34	1.44
50	3.09±0.08	5.86±0.03	4.96±0.14	59.4±1.2	------	9.88	4.16	1.40
55	2.74±0.008	5.52±0.002	5.32±0.2	60.8±1.4	------	9.94	4.18	1.56
60	2.56±0.02	5.46±0.10	6.90±0.20	78.5±2.7	------	6.12	3.57	1.23
ca. 68	Sphere (ellipsoid) – rod transition							
70	2.11±0.02	4.95±0.10	9.3±0.27	45.8±4.0	278±11	7.31	3.58	1.41
80	1.99±0.02	4.73±0.10	12.4±0.3	42.9±3.3	304±10	8.73	3.91	1.88
90	1.80±0.01	4.48±0.09	14.4±0.3	44.7±2.6	358±11	7.85	3.71	1.87
100	1.70±0.013	4.35±0.09	16.4±0.4	43.8±2.5	441±14	7.53	3.63	1.96

Table S5. Parameters of T = 28.9 °C determined with corresponding form factor. N_{C2H6} (900 bar) = 10.27×10^{21} cm^{-3}

Pressure [bar]	S(0) [cm^{-1}]	ξ [Å]	🖰dΣ/dw(0) [cm^{-1}]	R [Å]	L [Å]	Q2 [10^{-6} cm^{-1} $Å^{-3}$]	$\Phi^{1/2}\times$🖰n [10^{19} cm^{-3}]	Φ [10^{-4}]
36.6	0.341±0.008	6.21±0.031	-----	-----	-----	-----	-----	-----
41.7	1.37±0.002	12.5±0.038	-----	-----	-----	-----	-----	-----
44.1	gas-liquid line							
51.8	0.735±0.002	8.19±0.048	-----	-----	-----	-----	-----	-----
53.5±2.5	Frenkel line							
56.8	0.528±0.003	6.35±0.06	0.066±0.003	57.6±1.7	-----	1.33	5.45	2.25
61.8	0.46±0.003	6.33±0.076	0.086±0.002	75.3±1.4	-----	0.827	4.39	1.61
71.8	0.375±0.003	5.85±0.08	0.114±0.003	81.8±1.3	-----	0.863	4.78	2.23
76.8±5	Sphere (ellipsoid) – rod transition							
81.8	0.321±0.003	5.44±0.10	0.191±0.006	54.7±3.8	298.8±14.8	2.49	5.64	3.55
91.8	0.285±0.003	5.11±0.11	0.261±0.008	51.2±3.2	410.6±17.4	1.896	6.42	5.12
101.8	0.264±0.003	4.92±0.11	0.332±0.012	51.1±3.1	557.8±27	1.76	5.71	4.47
121.8	0.231±0.002	4.74±0.11	0.388±0.08	49±2.3	841±184	1.37	4.19	2.85

Table S6. Parameters of T = 33.2 °C determined with corresponding form factor. N_{C2H6} (900 bar) = 10.20×10^{21} cm^{-3}

Pressure [bar]	S(0) [cm^{-1}]	ξ [Å]	🖰dΣ/dw(0) [cm^{-1}]	R [Å]	L [Å]	Q2 [10^{-6} cm^{-1} $Å^{-3}$]	$Φ^{1/2}$×🖰n [10^{19} cm^{-3}]	Φ [10^{-4}]
36.6	0.217±0.0006	4.97±0.04	-----	-----	-----	-----	-----	-----
42.4	0.404±0.001	6.51±0.033	-----	-----	-----	-----	-----	-----
48.7±0.1	Widom line							
51.9	1.65±0.003	12.1±0.04	-----	-----	-----	-----	-----	-----
56.8	0.819±0.002	8.22±0.04	-----	-----	-----	-----	-----	-----
59.3±2.5	Frenkel line							
61.8	0.59±0.008	6.72±0.17	0.062±0.002	61.3±2.9	-----	1.074	4.33	1.32
71.8	0.434±0.004	5.99±0.13	0.085±0.002	73.9±1.5	-----	0.868	3.90	1.30
77±5	Sphere (ellipsoid) – rod transition							
81.8	0.365±0.003	5.67±0.10	0.136±0.005	51.8±4.9	219±14	1.83	5.66	3.17
91.8	0.317±0.003	5.21±0.10	0.192±0.005	49.8±1.7	264±12	1.9	5.77	3.73
101.8	0.287±0.002	5.11±0.10	0.245±0.006	50.8±2.7	358±12.5	1.95	5.84	4.25
121.8	0.247±0.002	4.82±0.09	0.599±0.10	50.9±2.8	1146±210	1.53	5.18	4.02

Table S7. Parameters of T = 39.5 °C determined with corresponding form factor. N_{C2H6} (900 bar) = 10.11×10^{21} cm^{-3}

Pressure [bar]	S(0) [cm^{-1}]	ξ [Å]	🖰dΣ/dw(0) [cm^{-1}]	R [Å]	L [Å]	Q2 [10^{-6} cm^{-1} $Å^{-3}$]	$\Phi^{1/2}$×🖰n [10^{19} cm^{-3}]	Φ [10^{-4}]
36.6	0.159±0.001	4.48±0.11	-----	-----	-----	-----	-----	-----
42.4	0.24±0.001	5.29±0.07	-----	-----	-----	-----	-----	-----
51.8	0.98±0.002	9.82±0.05	-----	-----	-----	-----	-----	-----
57.3±0.3	Widom line							
56.8			-----	-----	-----	-----	-----	-----
61.8	1.175±0.002	9.75±0.04	-----	-----	-----	-----	-----	-----
66.8±5	Frenkel line							
71.8	0.641±0.002	7.58±0.05	0.0473±0.006	81±6.4	-----	0.367	2.57	0.44
81.8	0.459±0.004	6.2±0.099	0.0798±0.003	72.6±2	-----	0.859	3.88	1.22
86.8±5	Sphere (ellipsoid) – rod transition							
91.8	0.383±0.004	5.77±0.097	0.113±0.005	55.5±7.41	164.6±21.5	2.75	5.66	3.02
101.8	0.332±0.003	5.40±0.097	0.153±0.005	53.7±4.21	219.5±13.1	2.38	6.46	4.45
121.8	0.278±0.003	5.066±0.098	0.260±0.006	51.7±2.74	384.9±14.2	2.04	5.98	4.69

Table S8. Parameters of T = 43.2 °C determined with corresponding form factor. N_{C2H6} (900 bar) = 10.07×10^{21} cm^{-3}

Pressure [bar]	S(0) [10^{-1} cm^{-1}]	ξ [Å]	🖰dΣ/dw(0) [10^{-2} cm^{-1}]	R [Å]	Q2 [10^{-7} cm^{-1} Å^{-3}]	Φ$^{1/2}$×🖰n [10^{19} cm^{-3}]	Φ [10^{-4}]	R (dΣ/dw(0)/Q2)
36.6	1.46±0.004	3.97±0.07	-----	-----	-----	-----		-----
42	2.10±0.006	4.56±0.06	-----	-----	-----	-----		-----
46.8	3.30±0.006	5.62±0.03	-----	-----	-----	-----		-----
51.8	6.00±0.01	7.49±0.03	-----	-----	-----	-----		-----
56.8	12.50±0.03	10.96±0.03	-----	-----	-----	-----		-----
**60**	Widom linie							
61.8	13.40±0.03	11.3±0.04	-----	-----	-----	-----		-----
**67.7**	Frenkel line							
71.8	6.57±0.03	7.73±0.05	3.10±1.30	76.6±12	2.98	2.286	0.29	78.9
81.8	4.67±0.02	6.50±0.05	5.19±0.80	72.6±5.0	5.84	3.20	0.73	74.8
91.8	3.84±0.02	5.95±0.05	6.08±0.50	64.5±3.1	9.62	4.108	1.42	66.8
**94**	Sphere (ellipsoid) – rod transition							
101.8	3.40±0.01	5.68±0.05	9.92±0.7	77.0±2.7	9.33	4.046	1.57	79.4
111.8	3.16±0.01	5.42±0.06	11.40±0.7	77.0±2.4	12.8	4.739	2.43	72.9
121.8	2.99±0.01	5.15±0.05	10.50±0.40	68.5±1.6	10.71	4.334	2.25	77.3
145	1.44±0.01	4.89±0.07	6.44±0.40	72.2±2.4	7.33	3.586	1.90	74.5
173.1	2.84±0.01	4.50±0.07	9.10±0.60	72.1±2.5	10.39	4.269	3.36	74.5

3. Isolated droplets or randomly distributed nonparticulate two-phase morphology?

The scattering theories for thermal density fluctuations and isolated droplets with low concentration are listed in Appendix A. Both theories describe our SANS data very well. However, the droplet structure could also be viewed as a statistically random distribution of a non-particulate two-phase system whose scattering function was formulated by Debye-Anderson-Brumberger (DAB)[1,2] as described in detail by Hashimoto.[3] In this section, an analysis of the $\Delta d\Sigma/d\Omega(Q)$ measured at T = 28.9 ˚C and a pressure of 121 bar is carried out using the form factor of "isolated droplets" (i.e. randomly oriented elongated ellipsoids and rods) and the DAB model and comparing the results with respect to the reduced chi-squared statistics and Q_F analysis.

In our case, the DAB theory should describe the morphology of (non-thermal) density fluctuations following the spatial correlation function $\gamma(r) = \exp\left(-r/\xi_D\right)$ and the scattering law in eq S1 with the correlation distance ξ_D and the scattering $\Delta d\Sigma/d\Omega(0)$ at Q = 0. $\Delta d\Sigma/d\Omega(0)$ is

$$\Delta d\Sigma/d\Omega(Q) = \Delta d\Sigma/d\Omega(0)/\left(1+\xi_D^2 Q^2\right)^{\alpha} \text{ with } \alpha=2 \text{ and } \Delta d\Sigma/d\Omega(0) = 8\pi\,\Phi\left(1-\Phi\right)\xi_D^3\,\Delta\rho^2 \quad \text{(S1)}$$

determined by the product of $\Phi_A\Phi_B = \Phi(1\text{-}\Phi)$ $(\Phi = \Phi_A)$ of droplet volume fraction and their scattering contrast, $\Delta\rho^2$. For $\alpha = 1$, eq S1 corresponds to the Ornstein-Zernicke equation introduced in Eq. A1 of the main text.

Figure S7 shows $\Delta d\Sigma/d\Omega(Q)$ measured at T = 28.9 ˚C and P = 121.8 bar together with the fits of the various scattering laws. Table S9 compiles the structural parameter of the droplet phase together with the χ^2, R^2 and the Q Factor depicted as solid, dashed and dashed dotted lines. (ref.[4] section 2.7) Fitting the four models to $\Delta d\Sigma/d\Omega(Q)$ clearly favors the formation of elongated isolated droplets with ellipsoidal and rod-like shapes, with a clear preference for the rod-like structure.

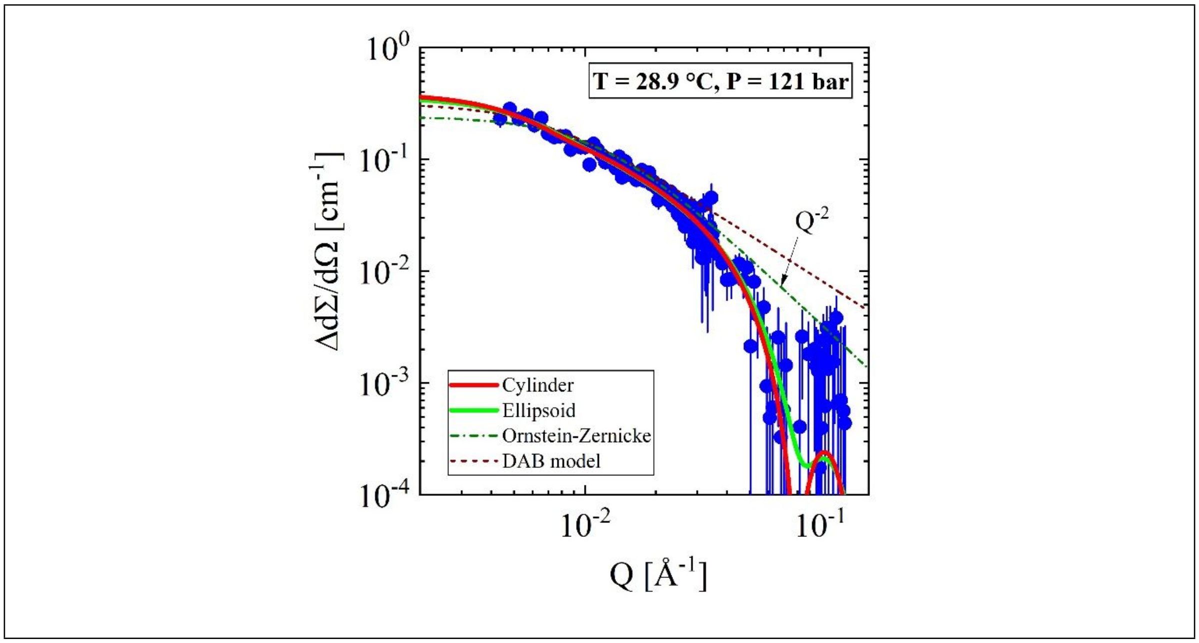

Figure S7. Plot of $\Delta d\Sigma/d\Omega(Q)$ at T = 28.9 °C and P = 121.8 bar of Figure 3 fitted with various model scattering laws.

Table S9. Fit parameters of $\Delta d\Sigma/d\Omega(Q)$ for various models at T = 28.9 °C and P = 121.8 bar

Model	$\Delta d\Sigma/d\Omega(0)$ [cm^{-1}]	R [Å]	Epsilon/ L [Å]	α	ξ_2	χ^2	R^2	Q-Factor
Ornstein-Zernicke	0.242±0.011	-----	-----	1	83.4 ± 3.8	1.34	0.996	4.6×10^{-3}
DAB model	0.347±0.053	-----	-----	0.68 ± 0.12	171 ± 46	1.28	0.997	0.016
Ellipsoid	0.36±0.06	50.2 ± 2.8	9.9 ± 1.9 (E)	-----	-----	1.114	1.715	0.172
Rod	0.388±0.08	49 ± 2.3	841 ± 185 (L)	-----	-----	1.073	0.997	0.266

References

(1) Debye, P.; Bueche, A. M. Scattering by an inhomogeneous solid. *J. Appl. Phys*. **1949**, *20*, 518–525.

(2) Debye, P.; Anderson, R.; Brumberger, H., Scattering by an Inhomogeneous Solid. II. The Correlation Function and Its Application, *J. Appl. Phys*. **1957**, *28* (6), 679-683.

(3) Hashimoto, T. *Principles and Applications of X-ray, Light and Neutron Scattering* Springer Verlag, Singapore 2023, 978-981-16-1647-1 (ISBN).

(4) Kohlbrecher, *J. SASfit: A program for fitting simple structural models to small angle scattering data,* Paul Scherrer Institute Laboratory for Neutron Scattering and Imaging (LNS) CH-5232 Villigen PSI, December **2023.**
